# Palladium-catalyzed solid-state borylation of aryl halides using mechanochemistry

**DOI:** 10.3762/bjoc.18.86

**Published:** 2022-07-18

**Authors:** Koji Kubota, Emiru Baba, Tamae Seo, Tatsuo Ishiyama, Hajime Ito

**Affiliations:** 1 Division of Applied Chemistry, Graduate School of Engineering, Hokkaido University, Sapporo, Hokkaido 060-8628, Japan; 2 Institute for Chemical Reaction Design and Discovery (WPI-ICReDD), Hokkaido University, Sapporo, Hokkaido 060-8628, Japan

**Keywords:** ball mill, borylation, cross-coupling, mechanochemistry, solid-state reaction

## Abstract

This study describes the solid-state palladium-catalyzed cross-coupling between aryl halides and bis(pinacolato)diboron using ball milling. The reactions were completed within 10 min for most aryl halides to afford a variety of synthetically useful arylboronates in high yields. Notably, all experimental operations could be performed in air, and did not require the use of large amounts of dry and degassed organic solvents. The utility of this method was further demonstrated by gram-scale synthesis under solvent-free, mechanochemical conditions.

## Introduction

Arylboronic acid and its derivatives are indispensable reagents in modern synthetic chemistry because they have been frequently used for the preparation of many bioactive molecules, natural products, and functional organic materials, typically through Suzuki–Miyaura coupling [1–7]. The palladium-catalyzed boryl substitution of aryl halides with boron reagents, termed Miyaura–Ishiyama borylation, is an efficient method for synthesizing arylboronates with high functional group compatibility [8–14]. To date, many palladium-based catalytic systems in solution for the borylation of aryl halides have been reported [8–14]. However, these solution-based reactions usually require long reaction times and significant amounts of dry and degassed organic solvents. Additionally, to avoid the deterioration of reactivity due to moisture and oxygen, conventional protocols require synthesis techniques that involve the use of high-vacuum Schlenk lines and/or glove boxes, which are costly and require special training to handle. Thus, the development of an operationally simple, solvent-free palladium-catalyzed borylation process applicable for a wide range of aryl halides would greatly improve the practicality of the desired arylboronic acid derivatives.

Nechaev et al. reported the first solvent-free protocol for the palladium-catalyzed Miyaura–Ishiyama borylation of aryl halides in a test tube [15]. They found that a Pd(dba)_2_/DPEphos catalytic system was effective for aryl bromides, and aryl chlorides reacted more efficiently when XPhos was used as the ligand [15]. Although their achievements are remarkable, this protocol is only applicable to liquid substrates, which can serve as reactants and solvents (neat liquid conditions). In addition, a long reaction time (12 h) is required to complete the reaction [15]. A general and efficient solvent-free borylation protocol that can be applied to liquid as well as solid aryl halides remains undeveloped.

**Scheme 1:**
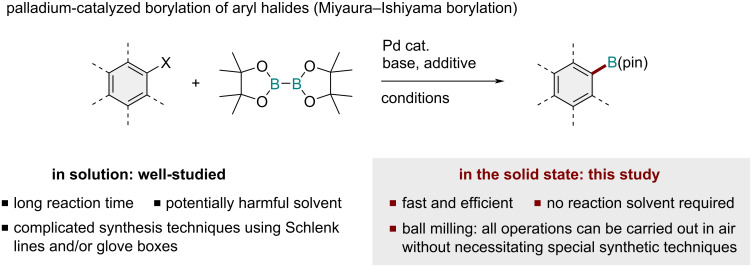
Development of the first solid-state palladium-catalyzed borylation protocol of aryl halides using mechanochemistry.

Recently, mechanochemical synthesis using ball milling has attracted considerable attention as an efficient solvent-free synthetic technique [16–33]. Notably, the strong mechanical agitation provided by ball milling enables efficient solid-state organic transformations. Thus far, mechanochemical palladium-catalyzed cross-coupling reactions such as Suzuki–Miyaura [34–47], Buchwald–Hartwig [48–52], Sonogashira [53–56], Negishi [57], Mizoroki–Heck [58–60], and C–S bond-forming [61] reactions have been developed. Our group has also been interested in this class of mechanochemical transformations, particularly in the development of cross-coupling reactions that proceed in the solid state [43–50]. These mechanochemical cross-coupling reactions often show much faster reaction kinetics than those under conventional solution-based conditions because of the high concentration; further, the experimental operations can be carried out in air. Considering these achievements, including our recent success in solid-state cross-coupling chemistry, we envisioned that this mechanochemical strategy could be applied to the palladium-catalyzed borylation of aryl halides [8–14]. The development of a solid-state borylation protocol could provide a practical solution to the many issues associated with conventional solution-based protocols (Scheme 1).

## Results and Discussion

Initially, we conducted an optimization study on the mechanochemical cross-coupling between 2-bromo-6-methoxynaphthalene (**1a**, 0.3 mmol) and bis(pinacolato)diboron (**2**, 1.2 equiv) in the presence of Pd(OAc)_2_ (2 mol %), KOAc (3.0 equiv), and H_2_O (60 μL) as a liquid additive [43–45] (Table 1). The reactions were conducted in a Retch MM400 ball mill in a stainless-steel milling jar (1.5 mL) at 30 Hz using one stainless-steel ball (diameter: 5 mm) for 10 min. We used a commercially available temperature-controllable heat gun that was placed directly above the ball-milling jar to control the reaction temperature [45]. Reactions were performed using a heat gun with a preset temperature of 100 °C, and the internal temperature of the reaction mixture (60 °C) was assessed by thermography immediately after opening the milling jar (see Supporting Information File 1 for details). First, we tested various phosphine ligands [62] for this reaction (Table 1, entries 1–8). Interestingly, we found that tri-*tert*-butylphosphonium tetrafluoroborate (*t*-Bu_3_P·HBF_4_) provided the borylation product **3a** in excellent yield (92%, Table 1, entry 1), with a small amount of the protonation product **4a** (5%, entry 1). When the reaction was stopped after 5 min, only a 26% yield of **3a** was obtained (Table 1, entry 2). The use of 1,1'-bis(diphenylphosphino)ferrocene (dppf), which is the optimal ligand under the original conditions for the solution-based protocol [8], provided only trace amounts of **3a** (1%, Table 1, entry 3). Reactions with DPEphos and XPhos, which are the optimal ligands for neat liquid conditions reported by Nechaev [15], also yielded only poor results (Table 1, entries 4 and 5). Other monophosphine ligands, such as SPhos, PCy_3_, and PAd_3_, did not improve the yield of **3a** (Table 1, entries 6–8). The amount of H_2_O added was important for the efficiency of the reaction. Decreasing the amount of H_2_O to 40 μL led to a lower yield of **3a** (68%; Table 1, entry 9). The reaction without H_2_O afforded **3a** in only 8% yield (Table 1, entry 10). Finally, we investigated the effect of the reaction temperature. The reaction at a higher temperature (110 °C) also provided **3a** in a good yield (90%; Table 1, entry 11), but when the reaction was carried out at 30 °C, the yield was very low (6%; Table 1, entry 12). A longer reaction time (90 min) at 30 °C afforded **3a** in a moderate yield (41%; Table 1, entry 13). Note that no homocoupling product of **1a** was formed, or only trace amounts (<1%) were formed in all cases.

**Table 1:** Optimization of the reaction conditions.^a^

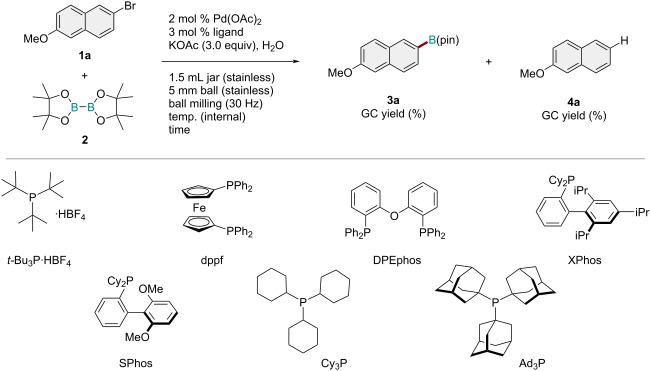						

Entry	Ligand	Amount of H_2_O (μL)	Internal temp (°C)	Time (min)	Yield of **3a** (%)^b^	Yield of **4a** (%)^b^

1	*t-*Bu_3_P·HBF_4_	60	60	10	92	5
2	*t-*Bu_3_P·HBF_4_	60	60	5	26	1
3	dppf	60	60	10	1	<1
4	DPEphos	60	60	10	<1	<1
5	XPhos	60	60	10	5	<1
6	SPhos	60	60	10	2	<1
7	PCy_3_	60	60	10	5	<1
8	PAd_3_	60	60	10	6	<1
9	*t-*Bu_3_P·HBF_4_	40	60	10	68	3
10	*t-*Bu_3_P·HBF_4_	0	60	10	8	1
11	*t-*Bu_3_P·HBF_4_	60	110	10	90	6
12	*t-*Bu_3_P·HBF_4_	60	30	10	6	1
13	*t-*Bu_3_P·HBF_4_	60	30	90	41	2

^a^Reaction conditions: a mixture of **1a** (0.30 mmol), **2** (0.36 mmol), KOAc (0.9 mmol), Pd(OAc)_2_ (0.006 mmol), ligand (0.009 mmol), and H_2_O was milled in a 1.5 mL stainless-steel jar at 30 Hz with one stainless-steel ball that was 5 mm in diameter. ^b^Determined by GC analysis using an internal standard.

**Scheme 2:**
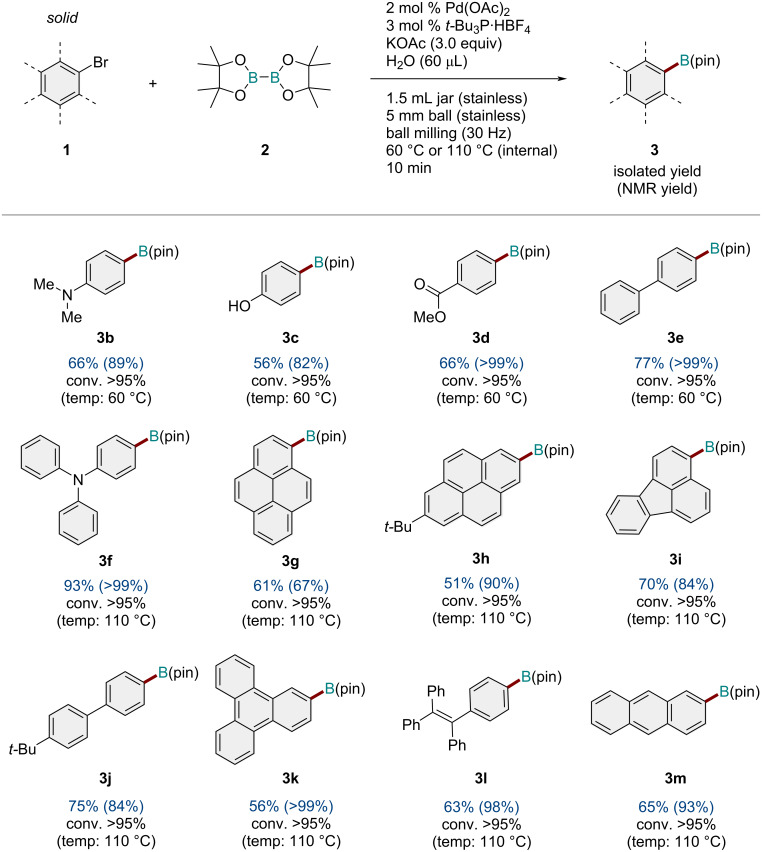
Substrate scope of solid aryl bromides. Reaction conditions: a mixture of **1** (0.30 mmol), **2** (0.36 mmol), KOAc (0.9 mmol), Pd(OAc)_2_ (0.006 mmol), *t-*Bu_3_·HBF_4_ (0.009 mmol), and H_2_O (60 μL) was milled in a 1.5 mL stainless-steel jar at 30 Hz using one stainless-steel ball that was 5 mm in diameter. The isolated yields are shown. The NMR yields are shown in parentheses.

Under the optimized reaction conditions (Table 1, entry 1), we explored the substrate scope of aryl bromides for the solid-state borylation (Scheme 2). The reactions of aryl bromides bearing electron-donating and electron-withdrawing groups at the *para* position (**1b**–**d**) proceeded smoothly to afford the corresponding arylboronates (**3b**–**d**) in good to high yields. 4-Bromobiphenyl (**1e**) also underwent borylation efficiently to form the borylation product (**3e**) in excellent yield. We found that substrates bearing relatively large conjugated structures (**1f**−**m**) tended to show low reactivity under the optimized conditions at 60 °C, but the reactions proceeded smoothly at 110 °C. For example, the bromo-substituted triphenylamine (**1f**), pyrene (**1g** and **1h**), fluoranthene (**1i**), biphenyl (**1j**), triphenylene (**1k**), tetraphenylethylene (**1l**), and anthracene (**1m**) reacted with diboron **2** to form the desired products (**3f**–**m**) in high yields.

**Scheme 3:**
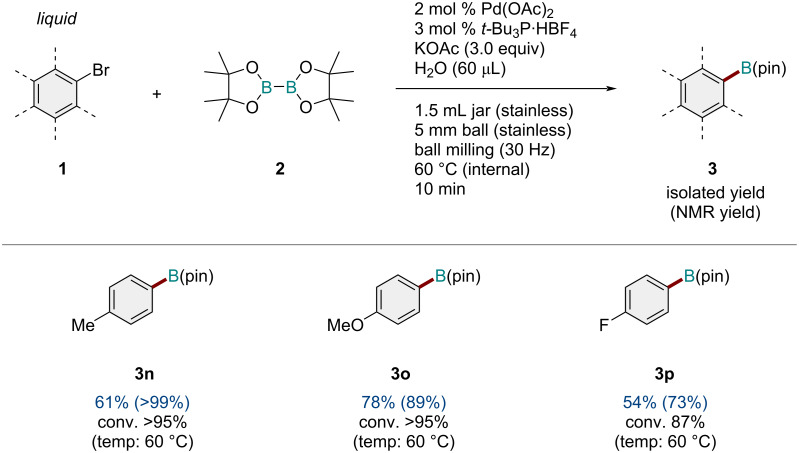
Substrate scope of liquid aryl bromides. Reaction conditions: a mixture of **1** (0.30 mmol), **2** (0.36 mmol), KOAc (0.9 mmol), Pd(OAc)_2_ (0.006 mmol), *t-*Bu_3_·HBF_4_ (0.009 mmol), and H_2_O (60 μL) was milled in a 1.5 mL stainless-steel jar at 30 Hz using one stainless-steel ball that was 5 mm in diameter. The isolated yields are shown. The NMR yields are shown in parentheses.

Next, the substrate scope of liquid aryl bromides was investigated (Scheme 3). We found that the present mechanochemical conditions were applicable to the solid substrate and various liquid substrates (**1n**–**p**), and the desired borylation products (**3n**–**p**) were obtained in good yields.

**Scheme 4:**
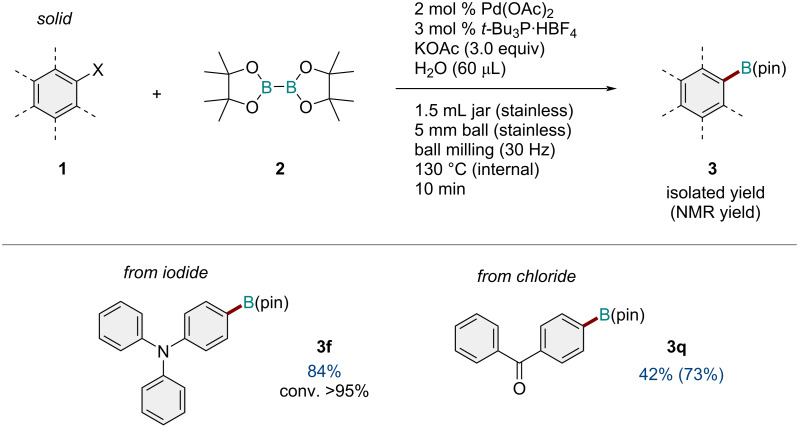
Reactions of solid aryl iodide and chloride. Reaction conditions: a mixture of **1** (0.30 mmol), **2** (0.36 mmol), KOAc (0.9 mmol), Pd(OAc)_2_ (0.006 mmol), *t-*Bu_3_·HBF_4_ (0.009 mmol), and H_2_O (60 μL) was milled in a 1.5 mL stainless-steel jar at 30 Hz using one stainless-steel ball that was 5 mm in diameter. The isolated yields are shown. The NMR yields are shown in parentheses.

We also investigated the solid-state borylation reactions of aryl iodides and chlorides (Scheme 4). The reaction of 4-iodo-*N*,*N*-diphenylaniline (**1q**) under the optimized conditions at 130 °C for 30 min proceeded smoothly and provided the desired product **3f** in high yield (84%). Also, the reaction of (4-chlorophenyl)(phenyl)methanone (**1r**) afforded the corresponding product **3q** in good yield.

**Scheme 5:**
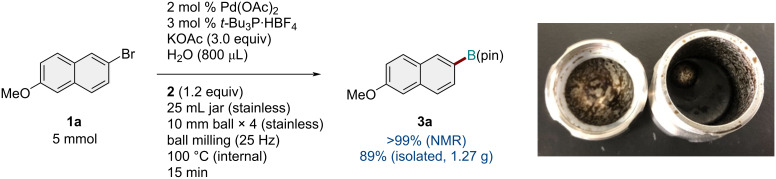
Solid-state borylation of aryl halides on a gram scale.

To demonstrate the practical utility of the developed protocol, the solid-state borylation reaction was investigated on a gram scale (Scheme 5). We carried out the reaction of **1a** at 5 mmol in the presence of the optimized catalytic system using a 25 mL stainless-steel jar and four stainless-steel balls (diameter: 10 mm) at 25 Hz and a preset heat-gun temperature of 150 °C. The gram-scale borylation proceeded efficiently and afforded the borylation product **3a** in high yield, which was comparable to the yield obtained at the 0.3 mmol scale. We confirmed by thermography that the internal temperature after the reaction was approximately 100 °C.

## Conclusion

In summary, we have developed the first protocol for the solid-state palladium-catalyzed borylation of aryl halides. This mechanochemical protocol allows the synthesis of various arylboronates in high yields within a short reaction time (within 10 min). Notably, the borylation reactions can be conducted without the use of organic solvents, and all synthetic operations can be carried out in air without the requirement of Schlenk-line techniques or glovebox operations. Therefore, the present solid-state approach is a practical and sustainable method to complement conventional solution-based protocols. The application of this method to the solid-state borylation of insoluble substrates for the synthesis of new arylboronates that are difficult to prepare by other means is currently under investigation.

## Supporting Information

File 1 Experimental procedures, experimental set-ups, characterization data, and NMR spectra.
