# Supplementary material for: Palladium-catalyzed solid-state borylation of aryl halides using mechanochemistry
Source: Beilstein J Org Chem. 2022 Jul 18;18:855–62. doi: 10.3762/bjoc.18.86 (PMC9344555; doi:10.3762/bjoc.18.86)

BEILSTEIN JOURNAL OF ORGANIC CHEMISTRY

# Supporting Information

for

# Palladium-catalyzed solid-state borylation of aryl halides using mechanochemistry

Koji Kubota, Emiru Baba, Tamae Seo, Tatsuo Ishiyama and Hajime Ito

*Beilstein J. Org. Chem.* **2022,** *18,* 855–862. doi:10.3762/bjoc.18.86

# Experimental procedures, experimental set-ups, characterization data, and NMR spectra

License and Terms: This is a supporting information file under the terms of the Creative Commons Attribution License (https://creativecommons.org/licenses/by/4.0). Please note that the reuse, redistribution and reproduction in particular requires that the author(s) and source are credited and that individual graphics may be subject to special legal provisions.
The license is subject to the *Beilstein Journal of Organic Chemistry* terms and conditions: (https://www.beilstein-journals.org/bjoc/terms)

## Table of contents

1. Chemicals and instrumentation ........................................................................................S2
2. General procedure for solid-state cross-coupling ......................................................................S4
3. Set-up procedure for high-temperature ball-milling reaction .................................................S5
4. Thermography observation for reaction temperature ...............................................................S7
5. Characterization of coupling products .......................................................................................S9
6. References ...................................................................................................................................S18
7. NMR Spectra ...............................................................................................................................S19

## 1. Chemicals and instrumentation

The starting materials were obtained from commercial suppliers and used as received. Bis(pinacolato)diboron [$B_2(pin)_2$] was recrystallized from pentane prior to use.[1] All mechanochemical reactions were carried out using grinding vessels in a Retsch MM400 mill (Figure S1). Both jars (1.5 mL) and balls (5 mm) are made of stainless steel (SUS400B and SUS420J2, respectively) (Figure S2). The heat gun Takagi HG-1450B with temperature control function was used for high-temperature ball-milling reactions (Figure S3). NMR spectra were recorded on JEOL JNM-EC X400P and JNM-ECS400 spectrometers ($^1$H: 392 or 401 MHz, $^{13}$C: 99 or 101MHz). Tetramethylsilane ($^1$H), $CDCl_3$ ($^{13}$C) were employed as standards, respectively. Multiplicity was recorded as follows: s = singlet, brs = broad singlet, d = doublet, t = triplet, q = quartet, quint = quintet, sept = septet, o = octet, m = multiplet. 1,1,2,2-Tetrachloroethane was used as an internal standard to determine NMR yields. Thermography was recorded with an NEC Avio Thermo GEAR G120. High-resolution mass spectra were recorded at the Global Facility Center, Hokkaido University.

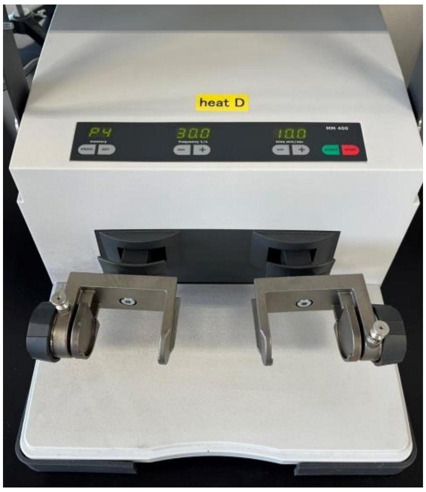


**Figure S1**. Retsch MM400 used in this study.

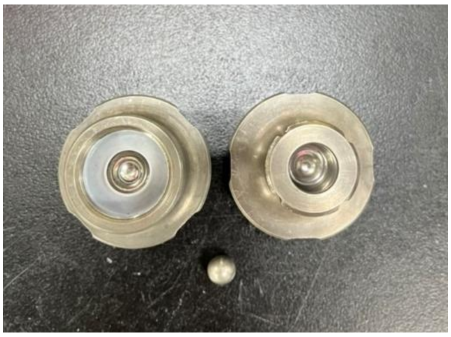

**Figure S2**. Stainless steel jar (1.5 mL) and ball (5 mm) used in this study.

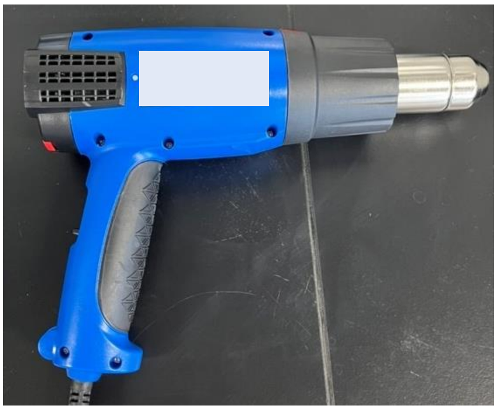

**Figure S3**. The temperature controllable heat gun Takagi HG-1450B used in this study.

## 2. General procedure for solid-state cross-coupling.

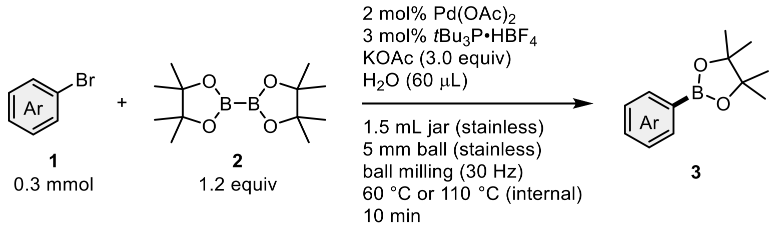


Aryl halide **1** (0.3 mmol), $B_2(pin)_2$ (0.36 mmol, 1.2 equiv), $Pd(OAc)_2$ (0.006 mmol, 2 mol %), *t*-$Bu_3 \cdot HBF_4$ (0.009 mmol, 3 mol %), and KOAc (0.9 mmol, 3.0 equiv) were placed in a ball-milling vessel (stainless steel, 1.5 mL) loaded with one grinding ball (stainless steel, diameter: 5 mm). Then, $H_2O$ (60 µL, 11 equiv) was added via syringe. After the vessel was closed in air without purging with inert gas, it was placed in the ball mill (Retsch MM400, 10 min or 30 min at 30 Hz) and heated with a heat gun (the preset temperature at 100 °C or 250 °C). After 10 min, the jar was opened after being cooled rapidly with cold water. The mixture was filtrated with $CH_2Cl_2/Et_2O$ 50% or EtOAc/$Et_2O$ 50% to remove the starting material and inorganic salts. The crude mixture was then purified by flash column chromatography ($SiO_2$, EtOAc/hexane, typically 0–10:90).

**For gram-scale reaction.**

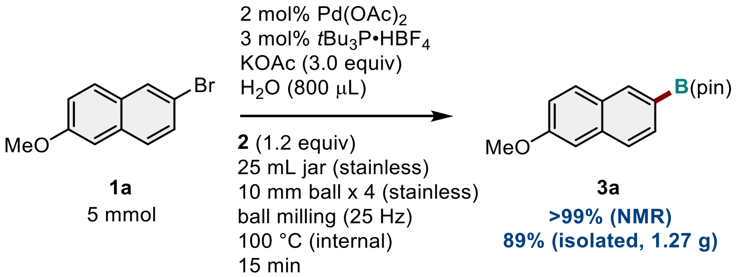


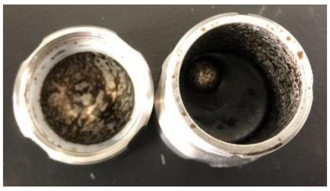

Aryl halide **1a** (1.185 g, 5.0 mmol), $B_2(pin)_2$ (1.524 g, 6.0 mmol, 1.2 equiv), $Pd(OAc)_2$ (22.5 mg, 0.10 mmol, 2 mol %), *t*-$Bu_3 \cdot HBF_4$ (43.5 mg, 0.15 mmol, 3 mol %), and KOAc (1.472 g, 15 mmol, 3.0 equiv) were placed in a ball-milling vessel (stainless steel, 25 mL) loaded with four grinding balls (stainless steel, diameter: 10 mm). Then, $H_2O$ (1.0 mL) was added via syringe. After the vessel was closed in air without purging with inert gas, it was placed in the ball mill (Retsch MM400, 15 min at 25 Hz) and heated by a heat gun (the preset temperature at 150 °C). The reaction was performed at 25 Hz because the use of the large jars at 30 Hz would overload the ball-mill equipment. After 15 min, the jar was opened after being cooled rapidly with cold water. The mixture was extracted with $CH_2Cl_2$ three times. Then, the organic layer was dried over $MgSO_4$, filtered, and concentrated in vacuo. The NMR yield was determined by $^1$H NMR analysis with an internal standard. The crude mixture was then purified by flash column chromatography ($SiO_2$, EtOAc/hexane, 0–10:90) to give **3a** in 89% yield (1.27 g).

## 3. Set-up procedure for high-temperature ball-milling reaction.

The heat gun was fixed with clamps and placed directly above the ball milling jar (distance between the heat gun and ball milling jar: ca. 1 cm) (Figure S4). The set-up procedure for high-temperature ball-milling reactions is shown in Figure S5. First, one grinding ball (stainless steel, diameter: 5 mm) was placed in the ball milling jar (stainless steel, 1.5 mL). Then, solid and liquid materials were added to the jar. After the ball milling jar was closed, the jar was placed in the ball mill (Retsch MM400), and a heat gun was placed directly above the ball-milling jar. The mechanochemical borylation reactions were conducted while applying heated air to the outside of the milling jar. When the preset temperature of the heat gun is set to 100 °C, for a 1.5 mL stainless jar at 30 Hz for 10 min, the internal temperature was determined to be 60 °C. When the preset temperature of the heat gun is set to 250 °C, for a 1.5 mL stainless steel jar at 30 Hz for 10 min, the internal temperature was determined to be 110 °C. When the preset temperature of the heat gun is set to 250 °C, for 1.5 mL stainless steel jar at 30 Hz for 30 min, the internal temperature was determined to be 130 °C. When the preset temperature of the heat gun is set to 150 °C, for a 25 mL stainless steel jar at 25 Hz for 15 min, the internal temperature was determined to be 100 °C.

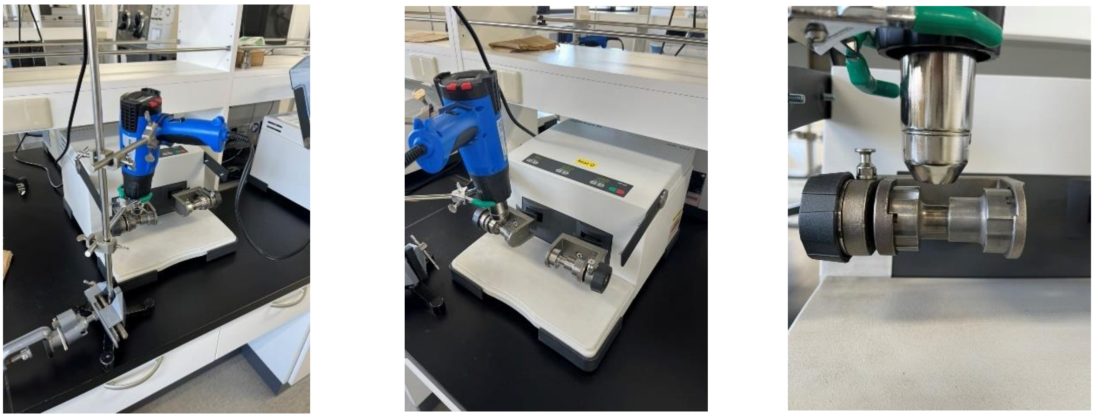

**Figure S4**. The set-up procedure for a heat gun on MM400.

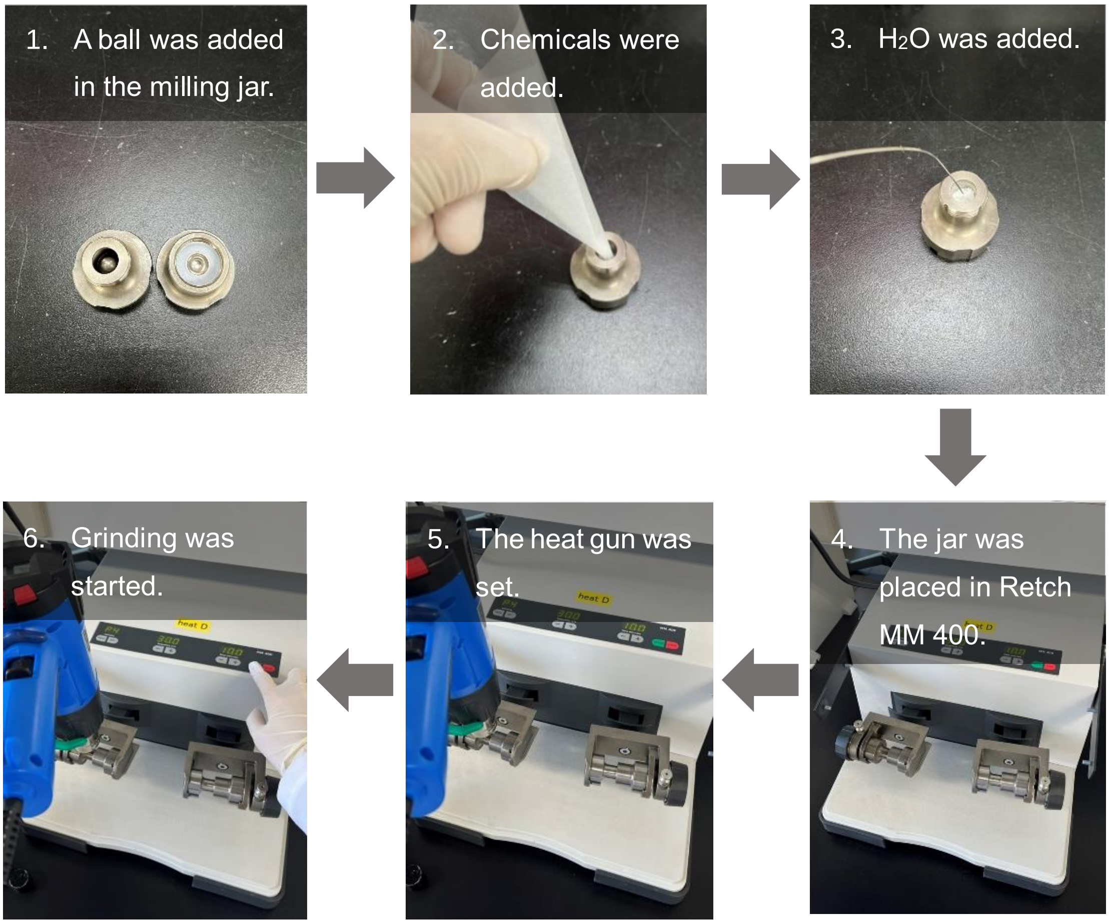


**Figure S5.** The set-up procedure for the high-temperature solid-state cross-coupling.

**4. Thermography observation for reaction temperature.**

The temperature inside the milling jar after the solid-state borylation reactions was confirmed by observation with a thermography camera immediately after opening the milling jar (Figures S6–S9).

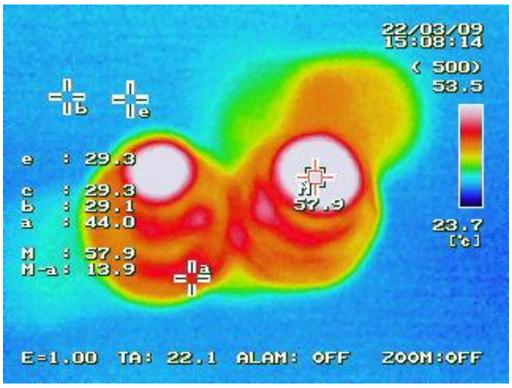


**Figure S6.** Thermographically derived temperature (57.9 °C) inside of the milling jar (1.5 mL) after grinding for 10 min at 30 Hz at a preset temperature of 100 °C.

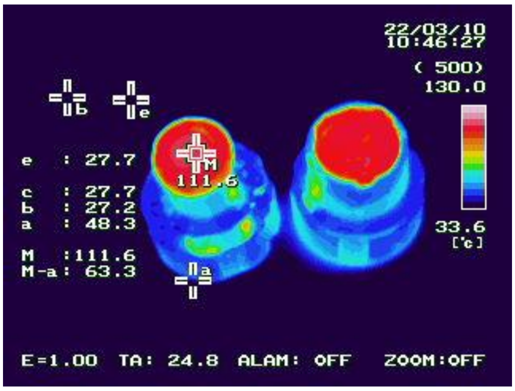


**Figure S7.** Thermographically derived temperature (111.6 °C) inside of the milling jar (1.5 mL) after grinding for 10 min at 30 Hz at a preset temperature of 250 °C.

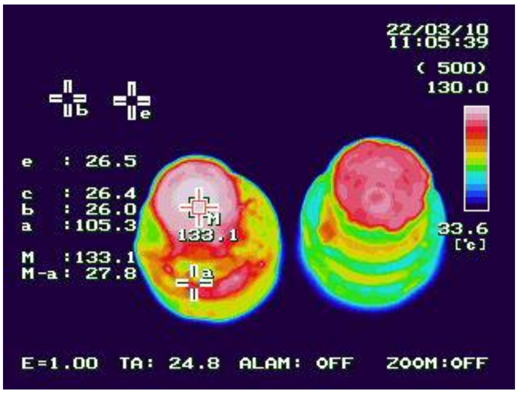


**Figure S8.** Thermographically derived temperature (133.1 °C) inside of the milling jar (1.5 mL) after grinding for 30 min at 30 Hz at a preset temperature of 250 °C.

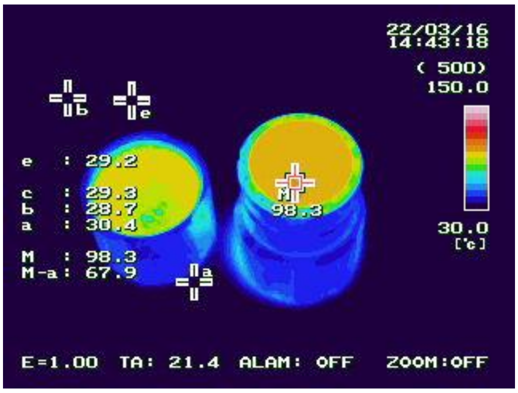


**Figure S9.** Thermographically derived temperature (98.3 °C) inside of the milling jar (25 mL) after grinding for 15 min at 25 Hz at a preset temperature of 150 °C.

## 5. Characterization of coupling products.

**2-(6-Methoxynaphthalen-2-yl)-4,4,5,5-tetramethyl-[1,3,2]dioxaborolane (3a).**

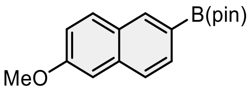


**3a**

The reaction was carried out with 71.1 mg (0.3 mmol) of **1a**. The product **3a** was obtained as a white solid (57.3 mg, 0.202 mmol, 67% yield) after purification by silica-gel column chromatography ($SiO_2$, EtOAc/hexane, 0:100–8:92). $^1H$ and $^{13}C$ NMR were in agreement with the literature.[2]

$^1H$ NMR (392 MHz, $CDCl_3$, δ): 1.38 (s, 12H), 3.90 (s, 3H), 7.09–7.15 (m, 2H), 7.71 (d, $J$ = 8.2 Hz, 1H), 7.77 (d, $J$ = 8.6 Hz, 1H), 7.81 (dd, $J$ = 1.0 Hz, 8.0 Hz, 1H), 8.29 (s, 1H). $^{13}C$ NMR (99 MHz, $CDCl_3$, δ): 25.0 ($CH_3$), 55.4 ($CH_3$), 83.9 ($C$), 105.7 ($C$H), 118.8 ($C$H), 126.0 ($C$H), 128.5 ($C$), 130.4 ($C$H), 131.2 ($C$H), 136.1 ($C$H), 136.5 ($C$), 158.6 ($C$). The carbon directly attached to the boron atom was not detected, likely due to quadrupolar relaxation. HRMS-EI (*m*/*z*): $[M]^+$ calcd for $C_{17}H_{21}{}^{10}BO_3$, 283.1620; found, 283.1613.

***N*,*N*-Dimethyl-4-(4,4,5,5-tetramethyl-1,3,2-dioxaborolan-2-yl)aniline (3b).**

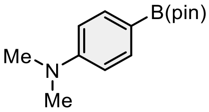


**3b**

The reaction was carried out with 60.0 mg (0.3 mmol) of **1b**. The product **3b** was obtained as white solid (49.2 mg, 0.199 mmol, 66% yield) after purification by silica-gel column chromatography ($SiO_2$, EtOAc/hexane, 1:99–6:94). $^1H$ and $^{13}C$ NMR were in agreement with the literature.[2]

$^1H$ NMR (392 MHz, $CDCl_3$, δ): 1.32 (s, 12H), 2.99 (s, 6H), 6.69 (d, $J$ = 9.0 Hz, 2H), 7.69 (d, $J$ = 8.6 Hz, 2H). $^{13}C$ NMR (99 MHz, $CDCl_3$, δ): 25.0 ($CH_3$), 40.2 ($CH_3$), 83.3 ($C$), 111.3 ($C$H), 136.2 ($C$H), 152.6 ($C$). The carbon directly attached to the boron atom was not detected, likely due to quadrupolar relaxation. HRMS-ESI (*m*/*z*): $[M+H]^+$ calcd for $C_{14}H_{23}O_2N^{10}B$, 247.1853; found, 247.1853.

**4-(4,4,5,5-Tetramethyl-1,3,2-dioxaborolan-2-yl)phenol (3c).**

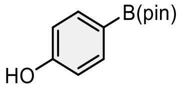


**3c**

The reaction was carried out with 51.8 mg (0.3 mmol) of **1c**. The product **3c** was obtained as a white solid (36.9 mg, 0.168 mmol, 56% yield) after purification by silica-gel column chromatography ($SiO_2$, EtOAc/hexane, 0:100–12:88). $^1$H and $^{13}$C NMR were in agreement with the literature.[3]

$^1$H NMR (392 MHz, $CDCl_3$, δ): 1.33 (s, 12H), 5.02 (s, 1H), 6.83 (d, $J$ = 11.2 Hz, 2H), 7.71 (d, $J$ = 8.0 Hz, 2H). $^{13}$C NMR (99 MHz, $CDCl_3$, δ): 24.9 ($C$H3), 84.0 ($C$), 115.1 ($C$H), 136.9 ($C$H), 158.7 ($C$). The carbon directly attached to the boron atom was not detected, likely due to quadrupolar relaxation. HRMS-EI ($m/z$): [M]$^+$ calcd for $C_{12}H_{17}{}^{10}BO_3$, 219.1307: found, 219.1299.

**Methyl 4-(4,4,5,5-tetramethyl-1,3,2-dioxaborolan-2-yl)benzoate (3d).**

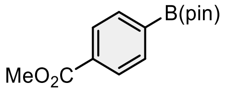


**3d**

The reaction was carried out with 64.6 mg (0.3 mmol) of **1d**. The product **3d** was obtained as a whie solid (51.9 mg, 0.198 mmol, 66% yield) after purification by silica-gel column chromatography ($SiO_2$, EtOAc/hexane, 0:100–9:91). $^1$H and $^{13}$C NMR were in agreement with the literature.[3]

$^1$H NMR (392 MHz, $CDCl_3$, δ): 1.36 (s, 12H), 3.92 (s, 3H), 7.87 (d, $J$ = 8.6 Hz, 2H), 8.02 (d, $J$ = 8.2 Hz, 2H).$^{13}$C NMR (99 MHz, $CDCl_3$, δ): 25.0 ($C$H3), 52.3 ($C$H3), 84.3 ($C$), 128.7 ($C$H), 132.4 ($C$), 134.8 ($C$H), 167.2 ($C$). The carbon directly attached to the boron atom was not detected, likely due to quadrupolar relaxation. HRMS-EI ($m/z$): [M]$^+$ calcd for $C_{14}H_{19}{}^{10}BO_4$, 261.1413; found, 261.1408.

**2-([1,1'-Biphenyl]-4-yl)-4,4,5,5-tetramethyl-1,3,2-dioxaborolane (3e).**

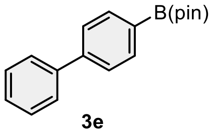


**3e**

The reaction was carried out with 69.9 mg (0.3 mmol) of **1e**. The product **3e** was obtained as a white solid (64.3 mg, 0.230 mmol, 77% yield) after purification by silica-gel column chromatography ($SiO_2$, EtOAc/hexane, 0:100–8:92). $^1$H and $^{13}$C NMR were in agreement with the literature.[2]

$^1$H NMR (392 MHz, $CDCl_3$, δ): 1.36 (s, 12H), 7.35 (tt, *J* = 1.6 Hz, 7.4 Hz, 1H), 7.44 (t, *J* = 7.3 Hz, 2H), 7.59–7.64 (m, 4H), 7.89 (d, *J* = 8.6 Hz, 2H). $^{13}$C NMR (99 MHz, $CDCl_3$, δ): 25.0 ($CH_3$), 83.9 (*C*), 126.6 (*C*H), 127.3 (*C*H), 127.7 (*C*H), 128.9 (*C*H), 135.4 (*C*H), 141.1 (*C*), 144.0 (*C*). The carbon directly attached to the boron atom was not detected, likely due to quadrupolar relaxation. HRMS-EI (*m*/*z*): $[M]^+$ calcd for $C_{18}H_{21}{}^{10}BO_2$, 279.1671; found, 279.1685.

***N*,*N*-Diphenyl-4-(4,4,5,5-tetramethyl-1,3,2-dioxaborolan-2-yl)aniline (3f).**

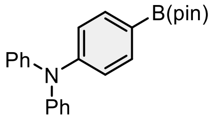


**3f**

**X = Br**: The reaction was carried out with 97.4 mg (0.3 mmol) of **1f** at 110 °C (The preset temperture was 250 °C) in 10 min. The product **3f** was obtained as a yellow oil (103.2 mg, 0.278 mmol, 93% yield) after purification by silica-gel column chromatography ($SiO_2$, EtOAc/hexane, 0:100–5:95).
**X = I**: The reaction was carried out with 111.4 mg (0.3 mmol) of **1f** at 130 °C (The preset temperture was 250 °C) in 30 min. The product **3f** was obtained as a yellow oil (93.9 mg, 0.253 mmol, 84% yield) after purification by silica-gel column chromatography ($SiO_2$, EtOAc/hexane, 0:100–5:95). $^1$H and $^{13}$C NMR were in agreement with the literature.[4]

$^1$H NMR (401 MHz, $CDCl_3$, δ): 1.33 (s, 12H), 7.01–7.06 (m, 4H), 7.10 (d, *J* = 7.6 Hz, 4H), 7.26 (t, *J* = 8.0 Hz, 4H), 7.66 (d, *J* = 8.8 Hz, 2H). $^{13}$C NMR (99 MHz, $CDCl_3$, δ): 25.0 ($CH_3$), 83.7 (*C*), 121.9 (*C*H), 123.5 (*C*H), 125.1 (*C*H), 129.4 (*C*H), 136.0 (*C*H), 147.5 (*C*), 150.7 (*C*). The carbon directly attached to the boron atom was not detected, likely due to quadrupolar relaxation. HRMS-EI (*m*/*z*): $[M]^+$ calcd for $C_{24}H_{26}{}^{10}BNO_2$, 370.2093; found, 370.2088.

**4,4,5,5-Tetramethyl-2-(pyren-1-yl)-1,3,2-dioxaborolane (3g).**

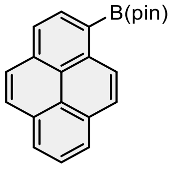


**3g**

The reaction was carried out with 84.3 mg (0.3 mmol) of **1g** at 110 °C (The preset temperture of the heat gun was 250 °C) in 10 min. The product **3g** was obtained as a yellow solid (59.9 mg, 0.183 mmol, 61% yield) after purification by silica-gel column chromatography ($SiO_2$, EtOAc/hexane, 1:99–6:94). $^1$H and $^{13}$C NMR were in agreement with the literature.[5]

$^1$H NMR (392 MHz, $CDCl_3$, δ): 1.49 (s, 12H), 8.00 (t, *J* = 7.6 Hz, 1H), 8.03–8.23 (m, 6H), 8.54 (d, *J* = 7.4 Hz, 1H), 9.07 (d, *J* = 9.0 Hz, 1H). $^{13}$C NMR (99 MHz, $CDCl_3$, δ): 25.2 (*C*H3), 84.0 (*C*), 124.2 (*C*H), 124.5 (*C*), 124.7 (*C*), 125.3 (*C*H), 125.5 (CH), 125.8 (*C*H), 127.6 (*C*H), 127.9 (*C*H), 128.1 (*C*H), 128.7 (*C*H), 130.9 (*C*), 131.2 (*C*), 133.6 (*C*), 134.0 (*C*H), 136.5 (*C*). The carbon directly attached to the boron atom was not detected, likely due to quadrupolar relaxation. HRMS-EI (*m*/*z*): $[M]^+$ calcd for $C_{22}H_{21}{}^{10}BO_2$, 327.1671; found, 327.1671.

**2-[7-(*tert*-Butyl)pyren-2-yl]-4,4,5,5-tetramethyl-1,3,2-dioxaborolane (3h).**

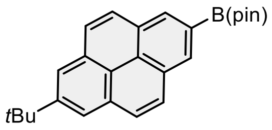


**3h**

The reaction was carried out with 101.3 mg (0.3 mmol) of **1h** at 110 °C (The preset temperture of the heat gun was 250 °C) in 10 min. The product **3h** was obtained as a white solid (58.4 mg, 0.152 mmol, 51% yield) after purification by silica-gel column chromatography ($SiO_2$, EtOAc/hexane, 1:99–6:94). $^1$H and $^{13}$C NMR were in agreement with the literature.[8]

$^1$H NMR (392 MHz, $CDCl_3$, δ): 1.46 (s, 12 H), 1.58 (s, 9 H), 8.05 (q, *J* = 9.0 Hz, 4H), 8.20 (s, 2H), 8.60 (s, 2H). $^{13}$C NMR (99 MHz, $CDCl_3$, δ): 25.1 (*C*H3), 32.1 (*C*H3), 35.4 (*C*), 84.2 (*C*), 122.2 (*C*H), 123.0 (*C*), 126.5 (*C*), 127.6 (*C*H), 127.8 (*C*H), 130.4 (*C*), 131.3 (*C*H), 131.6 (*C*), 149.7 (*C*). The carbon directly attached to the boron atom was not detected, likely due to quadrupolar relaxation. HRMS-EI (*m*/*z*): $[M]^+$ calcd for $C_{26}H_{29}{}^{10}BO_2$, 383.2297; found, 383.2294.

**2-(Fluoranthen-3-yl)-4,4,5,5-tetramethyl-1,3,2-dioxaborolane (3i).**

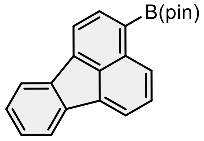

**3i**

The reaction was carried out with 84.3 mg (0.3 mmol) of **1i** at 110 °C (The preset temperture of the heat gun was 250 °C) in 10 min. The product **3i** was obtained as a yellow solid (69.1 mg, 0.211 mmol, 70% yield) after purification by silica-gel column chromatography ($SiO_2$, EtOAc/hexane, 1:99–6:94). $^1H$ and $^{13}C$ NMR were in agreement with the literature.[5]

$^1H$ NMR (392 MHz, $CDCl_3$, δ): 1.44 (s, 12H), 7.33–7.41 (m, 2H), 7.65 (dd, $J$ = 6.9 Hz, 8.4Hz, 1H), 7.86–7.96 (m, 4H), 8.22 (d, $J$ = 7.1 Hz, 1H), 8.60 (d, $J$ = 8.2 Hz, 1H). $^{13}C$ NMR (99 MHz, $CDCl_3$, δ): 25.1 ($CH_3$), 83.4 (*C*), 119.3 (*C*H), 120.1 (*C*H), 121.5 (*C*H), 122.0 (*C*H), 127.5 (*C*H), 128.2 (*C*H), 128.3 (*C*H), 128.4 (*C*H), 132.2 (*C*), 133.9 (*C*), 137.1 (*C*), 137.5 (*C*H) 139.2 (*C*), 140.0 (*C*), 140.1 (*C*). The carbon directly attached to the boron atom was not detected, likely due to quadrupolar relaxation. HRMS-EI (*m*/*z*): $[M]^+$ calcd for $C_{22}H_{21}{}^{10}BO_2$, 327.1671; found, 327.1676.

**2-[4'-(*tert*-Butyl)-[1,1'-biphenyl]-4-yl]-4,4,5,5-tetramethyl-1,3,2-dioxaborolane (3j).**

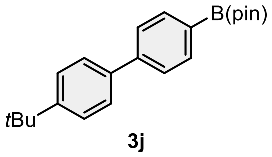

**3j**

The reaction was carried out with 86.9 mg (0.3 mmol) of **1j** at 110 °C (The preset temperture of the heat gun was 250 °C) in 10 min. The product **3j** was obtained as a brown solid (75.4 mg, 0.224 mmol, 75% yield) after purification by silica-gel column chromatography ($SiO_2$, EtOAc/hexane, 1:99–6:94). $^1H$ and $^{13}C$ NMR were in agreement with the literature.[3]

$^1H$ NMR (392 MHz, $CDCl_3$, δ): 1.36 (s, 21H), 7.47 (d, $J$ = 7.8Hz, 2H), 7.57 (d, $J$ = 8.6 Hz, 2H), 7.61 (d, $J$ = 6.3 Hz, 2H), 7.87 (d, $J$ = 8.62 Hz, 2H). $^{13}C$ NMR (99 MHz, $CDCl_3$, δ): 25.0 ($CH_3$), 31.5 ($CH_3$), 34.7 (*C*), 83.9 (*C*), 125.9 (*C*H), 126.4 (*C*H), 127.0 (*C*H), 135.4 (*C*H), 138.2 (*C*), 143.8 (*C*), 150.7 (*C*). The carbon directly attached to the boron atom was not detected, likely due to quadrupolar relaxation. HRMS-EI (*m*/*z*): $[M]^+$ calcd for $C_{22}H_{29}{}^{10}BO_2$, 335.2297; found, 335.2294.

**4,4,5,5-Tetramethyl-2-(triphenylen-2-yl)-1,3,2-dioxaborolane (3k).**

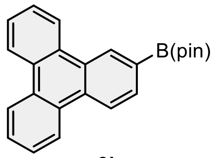


**3k**

The reaction was carried out with 92.3 mg (0.3 mmol) of **1k** at 110 °C (The preset temperture of the heat gun was 250 °C) in 30 min. The product **3k** was obtained as a white solid (59.7 mg, 0.169 mmol, 56% yield) after purification by silica-gel column chromatography ($SiO_2$, EtOAc/hexane, 1:99–6:94). $^1H$ and $^{13}C$ NMR were in agreement with the literature.[6]

$^1H$ NMR (392 MHz, $CDCl_3$, δ): 1.43 (s, 12H), 7.63–7.70 (m, 4H), 8.06 (d, $J$ = 8.2 Hz, 1H), 8.62–8.72 (m, 4H), 8.80–8.85 (m, 1H), 9.14 (s, 1H). $^{13}C$ NMR (99 MHz, $CDCl_3$, δ): 25.1 ($CH_3$), 84.1 (*C*), 122.6 (*C*H), 123.3 (*C*H), 123.4 (*C*H), 123.8 (*C*H), 127.28 (*C*H), 127.32 (*C*H), 127.7 (*C*H), 129.1 (*C*), 129.7 (*C*), 129.8 (*C*), 130.0 (*C*), 130.4 (*C*), 130.7 (*C*H), 132.1 (*C*), 132.9 (*C*H). The carbon directly attached to the boron atom was not detected, likely due to quadrupolar relaxation. HRMS-EI (*m*/*z*): $[M]^+$ calcd for $C_{24}H_{23}{}^{10}BO_2$, 353.1827; found, 353.1822.

**4,4,5,5-Tetramethyl-2-[4-(1,2,2-triphenylvinyl)phenyl]-1,3,2-dioxaborolane (3l).**

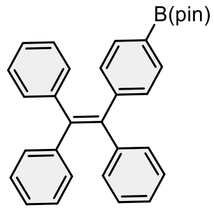


**3l**

The reaction was carried out with 123.4 mg (0.3 mmol) of **1l** at 110 °C (The preset temperture of the heat gun was 250 °C) in 10 min. The product **3l** was obtained as a white solid (87.2 mg, 0.190 mmol, 63% yield) after purification by silica-gel column chromatography ($SiO_2$, EtOAc/hexane, 1:99–6:94). $^1H$ and $^{13}C$ NMR were in agreement with the literature.[7]

$^1H$ NMR (392 MHz, $CDCl_3$, δ): 1.31 (s, 12H), 6.97–7.12 (m, 17H), 7.54 (d, $J$ = 8.6 Hz, 2H). $^{13}C$ NMR (99 MHz, $CDCl_3$, δ): 25.0 ($CH_3$), 83.8 (*C*), 126.56 (*C*H), 126.64 (*C*H), 127.75 (*C*H), 127.85 (*C*H), 130.8 (*C*H), 131.4 (*C*H), 131.5 (*C*H), 134.2 (*C*H), 140.9 (*C*), 141.5 (*C*), 143.6 (*C*), 143.7 (*C*), 143.8 (*C*), 146.9 (*C*). The carbon directly attached to the boron atom was not detected, likely due to quadrupolar relaxation. HRMS-EI (*m*/*z*): $[M]^+$ calcd for $C_{32}H_{31}{}^{10}BO_2$, 457.2453; found, 457.2434.

**2-(Anthracen-2-yl)-4,4,5,5-tetramethyl-1,3,2-dioxaborolane (3m).**

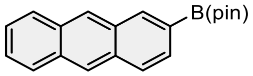


**3m**

The reaction was carried out with 77.2 mg (0.3 mmol) of **1m** at 110 °C (The preset temperture of the heat gun was 250 °C) in 10 min. The product **3m** was obtained as a white solid (59.8 mg, 0.197 mmol, 65% yield) after purification by silica-gel column chromatography ($SiO_2$, EtOAc/hexane, 1:99–10:90). $^1$H and $^{13}$C NMR were in agreement with the literature.[9]

$^1$H NMR (392 MHz, $CDCl_3$, δ): 1.41 (s, 12H), 7.43–7.51 (m, 2H), 7.79 (dd, $J$ = 1.2 Hz, 8.6 Hz, 1H), 7.97–8.03 (m, 3H), 8.40 (s, 1H), 8.47 (s, 1 H), 8.57 (s, 1H). $^{13}$C NMR (99 MHz, $CDCl_3$, δ): 25.1 ($CH_3$), 84.1 ($C$), 125.4 ($C$H), 125.9 ($C$H), 126.1 ($C$H), 127.3 ($C$H), 127.6 ($C$H), 128.3 ($C$H), 128.6 ($C$H), 129.3 ($C$H), 131.2 ($C$), 131.8 ($C$), 132.5 ($C$), 132.8 ($C$), 137.5 ($C$H). The carbon directly attached to the boron atom was not detected, likely due to quadrupolar relaxation. HRMS-EI ($m/z$): $[M]^+$ calcd for $C_{20}H_{21}{}^{10}BO_2$, 303.1671; found, 303.1666.

**4,4,5,5-Tetramethyl-2-(*p*-tolyl)-1,3,2-dioxaborolane (3n).**

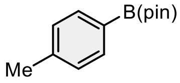


**3n**

The reaction was carried out with 51.7 mg (0.3 mmol) of **1n**. The product **3n** was obtained as a white solid (40.4 mg, 0.185 mmol, 61% yield) after purification by silica-gel column chromatography ($SiO_2$, EtOAc/hexane, 0:100–6:94). $^1$H and $^{13}$C NMR were in agreement with the literature.[2]

$^1$H NMR (392 MHz, $CDCl_3$, δ): 1.33 (s, 12H), 2.36 (s, 3H), 7.19 (d, $J$ = 7.1 Hz, 2H), 7.70 (d, $J$ = 7.1 Hz, 2H). $^{13}$C NMR (99 MHz, $CDCl_3$, δ): 21.9 ($CH_3$), 25.0 ($CH_3$), 83.7 ($C$), 128.6 ($C$H), 134.9 ($C$H), 141.5 ($C$). The carbon directly attached to the boron atom was not detected, likely due to quadrupolar relaxation. HRMS-EI ($m/z$): $[M]^+$ calcd for $C_{13}H_{19}{}^{10}BO_2$, 217.1514; found, 217.1513.

**2-(4-Methoxyphenyl)-4,4,5,5-tetramethyl-1,3,2-dioxaborolane (3o).**

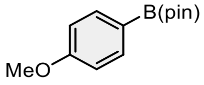


**3o**

The reaction was carried out with 55.9 mg (0.3 mmol) of **1o**. The product **3o** was obtained as a white solid (54.9 mg, 0.235 mmol, 78% yield) after purification by silica-gel column chromatography ($SiO_2$, EtOAc/hexane, 1:99–4:96). $^1$H and $^{13}$C NMR were in agreement with the literature.[2]

$^1$H NMR (392 MHz, $CDCl_3$, δ): 1.33 (s, 12H), 3.82 (s, 3H), 6.89 (d, $J$ = 9.0 Hz, 2H), 7.76 (d, $J$ = 8.6 Hz, 2H). $^{13}$C NMR (99 MHz, $CDCl_3$, δ): 25.0 (*C*$H_3$), 55.2 (*C*$H_3$), 83.7 (*C*), 113.4 (*C*H), 136.6 (*C*H), 162.2 (*C*). The carbon directly attached to the boron atom was not detected, likely due to quadrupolar relaxation. HRMS-EI (*m*/*z*): $[M]^+$ calcd for $C_{13}H_{19}{}^{10}BO_3$, 233.1464; found, 233.1460.

**2-(4-Fluorophenyl)-4,4,5,5-tetramethyl-1,3,2-dioxaborolane (3p).**

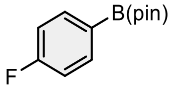


**3p**

The reaction was carried out with 52.6 mg (0.3 mmol) of **1p**. The product **3p** was obtained as a transparent oil (36.2 mg, 0.163 mmol, 54% yield) after purification by silica-gel column chromatography ($SiO_2$, EtOAc/hexane, 0:100–8:92). $^1$H and $^{13}$C NMR were in agreement with the literature.[3]

$^1$H NMR (392 MHz, $CDCl_3$, δ): 1.34 (s, 12H), 7.01–7.08 (m, 2H), 7.76–7.83 (m, 2H). $^{13}$C NMR (99 MHz, $CDCl_3$, δ): 25.0 (*C*$H_3$), 84.0 (*C*), 115.0 (d, $J_{C\text{-}F}$ = 19.8 Hz, *C*H), 137.1 (d, $J_{C\text{-}F}$ = 7.6 Hz, *C*H), 165.2 (d, $J_{C\text{-}F}$ = 250.3 Hz, *C*). The carbon directly attached to the boron atom was not detected, likely due to quadrupolar relaxation. HRMS-EI (*m*/*z*): $[M]^+$ calcd for $C_{12}H_{16}{}^{10}BFO_2$, 221.1264; found, 221.1274.

**Phenyl[4-(4,4,5,5-tetramethyl-1,3,2-dioxaborolan-2-yl)phenyl]methanone (3q).**

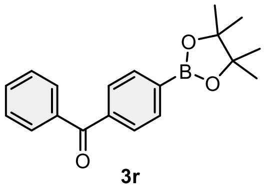

**3r**

The reaction was carried out with 64.9 mg (0.3 mmol) of **1r** at 130 °C (The preset temperture of the heat gun was 250 °C) in 30 min. The product **3q** was obtained as a white solid (38.8 mg, 0.126 mmol, 42% yield) after purification by silica-gel column chromatography ($SiO_2$, EtOAc/hexane, 1:99–4:96). $^{1}H$ and $^{13}C$ NMR were in agreement with the literature.[10]

$^{1}H$ NMR (401 MHz, $CDCl_3$, δ): 1.37 (s, 12H), 7.48 (t, $J$ = 7.6 Hz, 2H), 7.59 (t, $J$ = 7.6 Hz, 1H), 7.77 (d, $J$ = 8.4 Hz, 2H), 7.80 (d, $J$ = 7.2 Hz, 2H), 7.92 (d, $J$ = 8.0 Hz, 2H). $^{13}C$ NMR (101 MHz, $CDCl_3$, δ): 25.0 ($CH_3$), 84.3 ($C$), 128.4 ($C$H), 129.1 ($C$H), 130.2 ($C$H), 132.7 ($C$H), 134.7 ($C$H), 137.6 ($C$), 139.8 ($C$), 197.1 ($C$). The carbon directly attached to the boron atom was not detected, likely due to quadrupolar relaxation. HRMS-EI ($m/z$): $[M]^+$ calcd for $C_{19}H_{21}{}^{10}BO_3$, 307.1620; found, 307.1617.

## 6. References


1. Ishiyama, T.; Murata, M.; Ahiko, T.; Miyaura, N. *Org. Synth.* **2000**, *77*, 176–185.
2. Geng, S.; Zhang, J.; Chen, S.; Liu, Z.; Zeng, X.; He, Y.; Feng, Z. *Org. Lett.* **2020**, *22*, 5582–5588.
3. Ochiai, H.; Uetake, Y.; Niwa, T.; Hosoya, T. *Angew. Chem. Int. Ed.* **2017**, *56*, 2482–2486.
4. Liu, C.; Ji, C. L.; Hong, X.; Szostak, M. *Angew. Chem. Int. Ed.* **2018**, *57*, 16721–16726.
5. Fukazawa, M.; Takahashi, F.; Yorimitsu, H. *Org. Lett.* **2021**, *23*, 4613–4617.
6. Oda, S.; Ueura, K.; Kawakami, B.; Hatakeyama, T. *Org. Lett.* **2020**, *22*, 700–704.
7. Liang, A.; Wang, H.; Chen, Y.; Zheng, X.; Gao, T.; Yang, X.; Cai, P.; Wang, Z.; Zhang, F.; Huang, F. *Dyes and Pigments* **2018**, *149*, 399–406.
8. Bédard, A. C.; Vlassova, A.; Perez, A. C. H.: Bessette, A.; Hanan, G. S.; Heuft, M. A.; Collins, S. K. *Chem. Eur. J.* **2013**, *19*, 16295–16302.
9. Zeng, X.; Zhang, Y.; Liu, Z.; Geng, S.; He, Y.; Feng, Z. *Org. Lett.* **2020**, *22*, 29550–2955.
10. Tian, Y. M.; Guo, X. N.; Krummenacher, I.; Wu, Z.; Nitsch, J.; Braunschweig, H.; Radius, U. Marder, T. B. *J. Am. Chem. Soc.* **2020**, *142*, 18231–18242.

## 7. NMR Spectra

$^{1}$H NMR spectrum of **3a**.

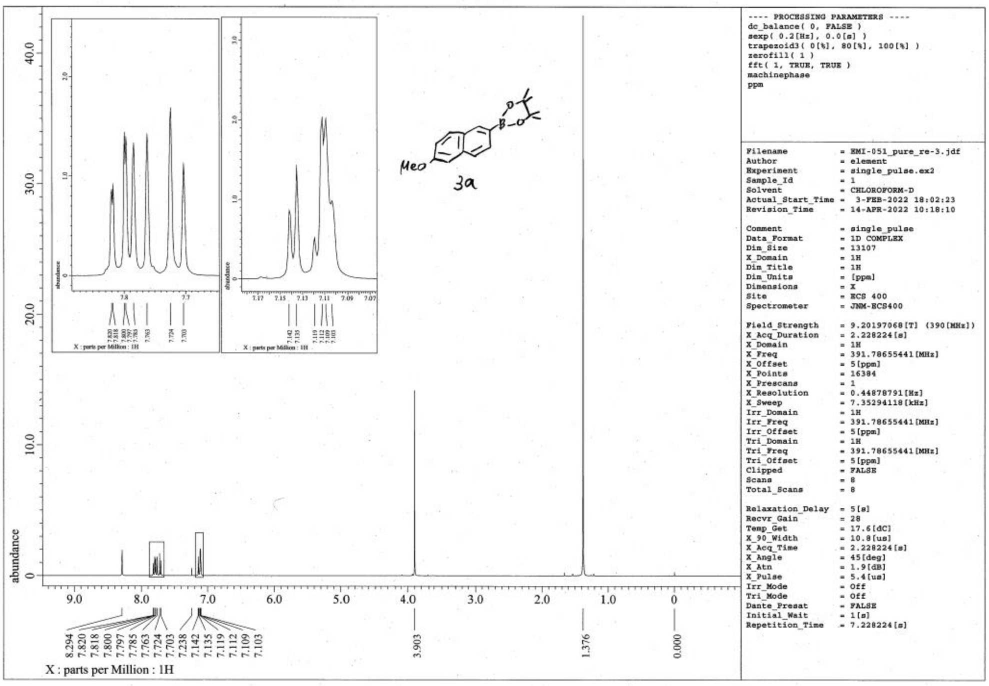


$^{13}$C NMR spectrum of **3a**.

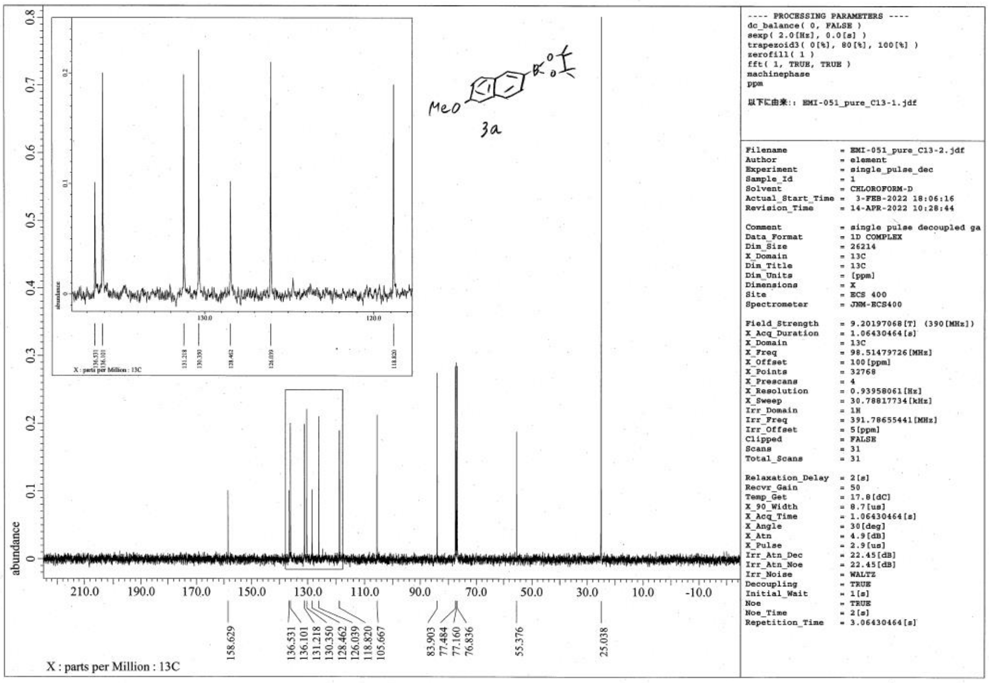

$^{1}H$ NMR spectrum of **3b**.

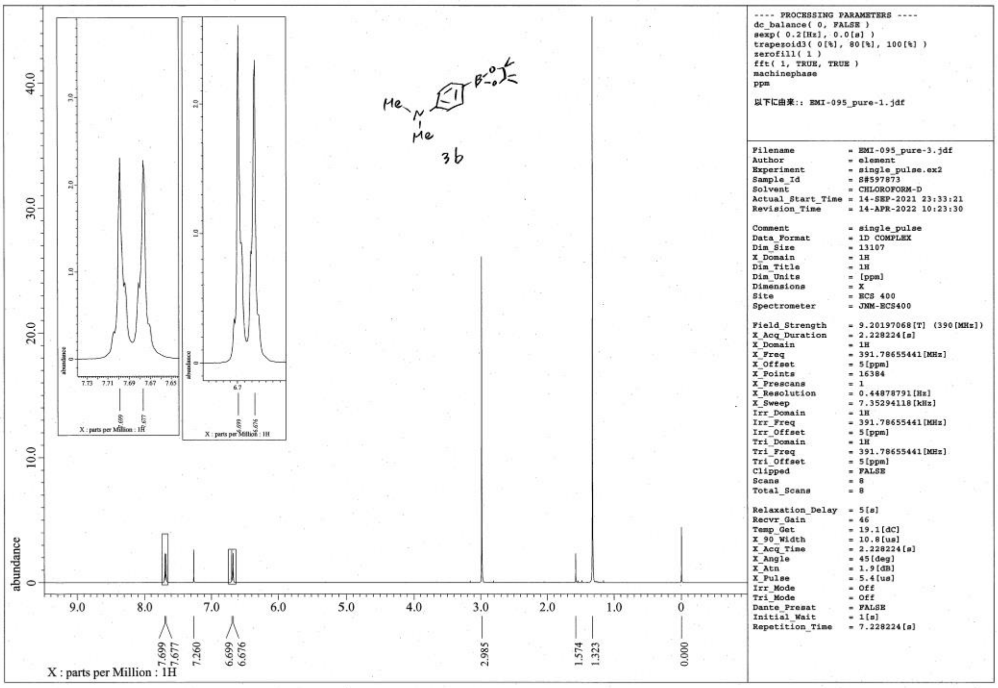

$^{13}C$ NMR spectrum of **3b**.

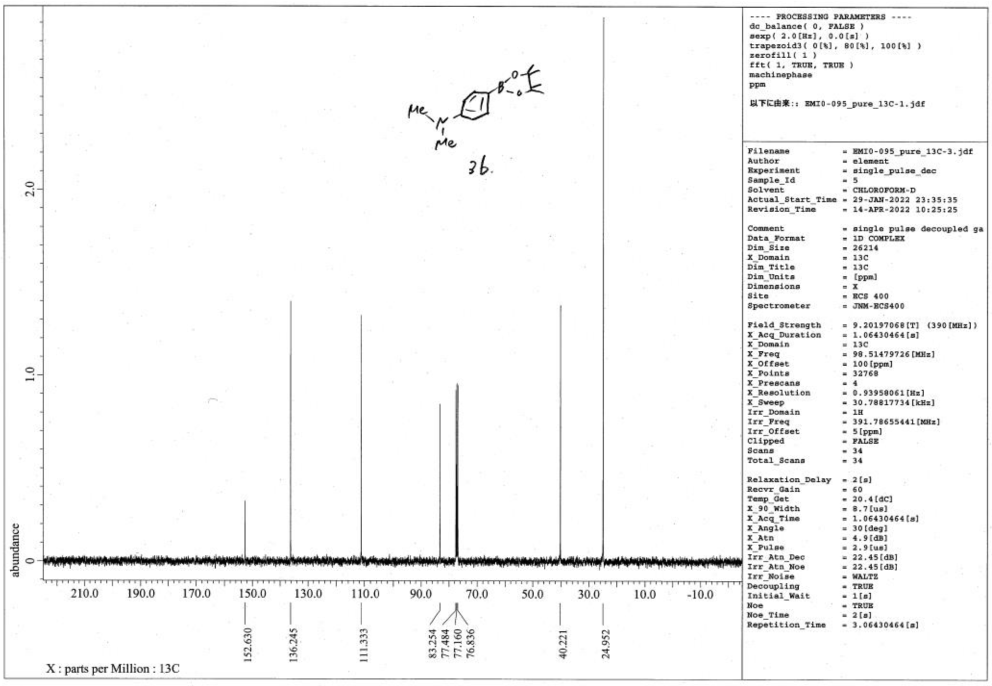

$^{1}$H NMR spectrum of **3c**.

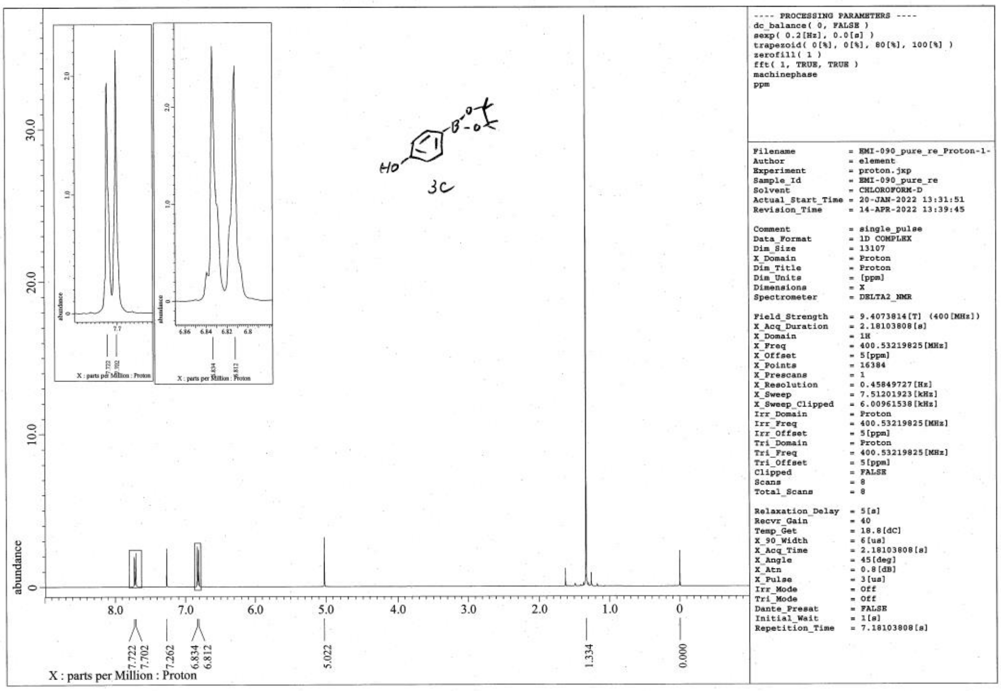

$^{13}$C NMR spectrum of **3c**.

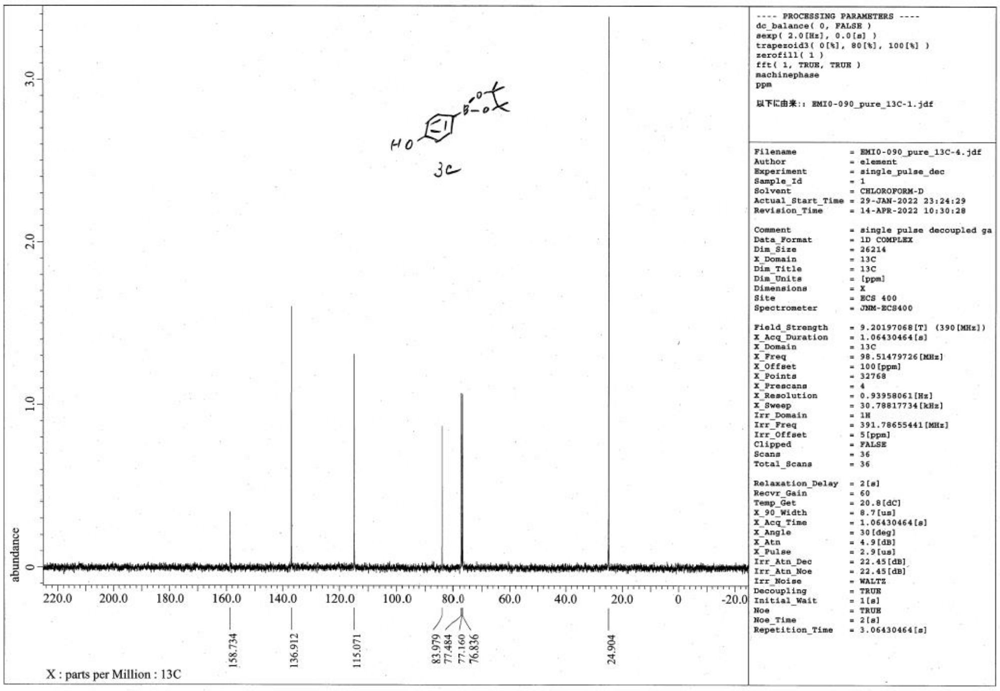

$^{1}$H NMR spectrum of **3d**.

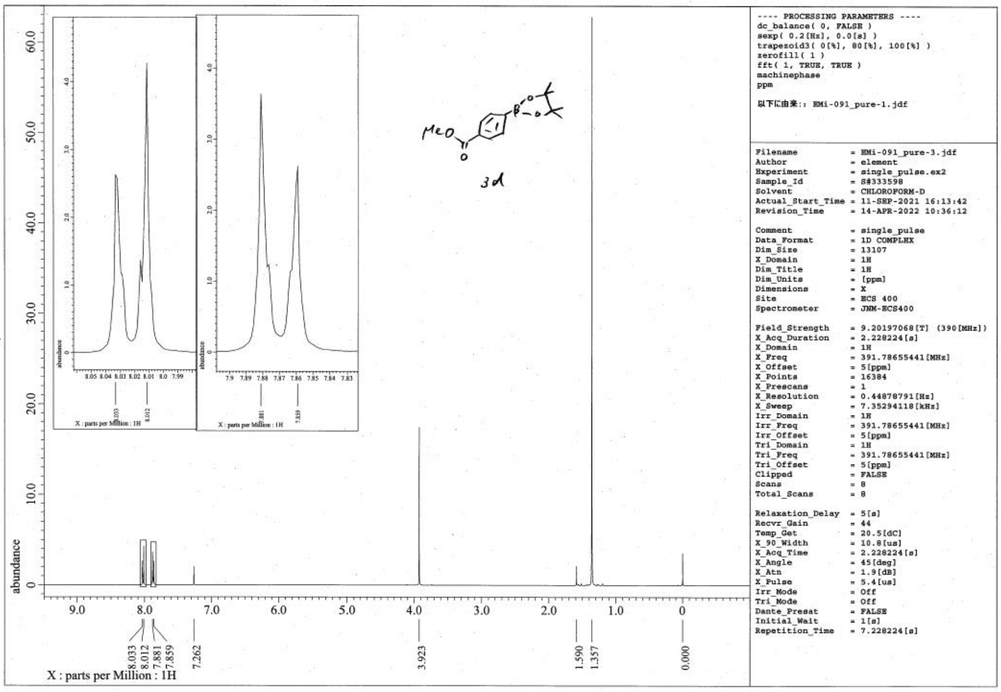

$^{13}$C NMR spectrum of **3d**.

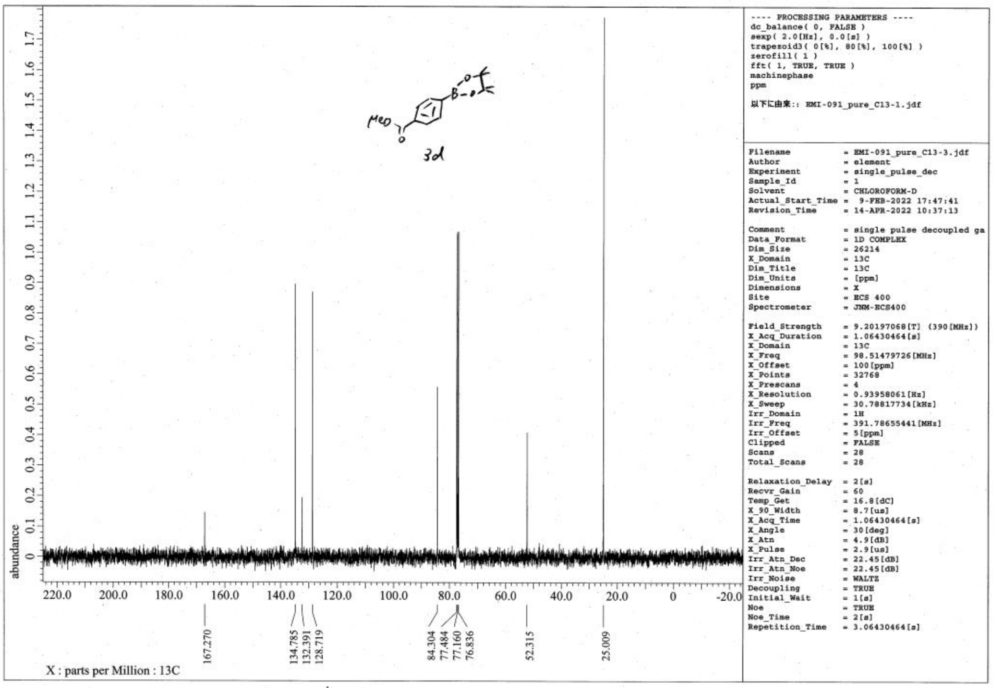

$^{1}$H NMR spectrum of **3e**.

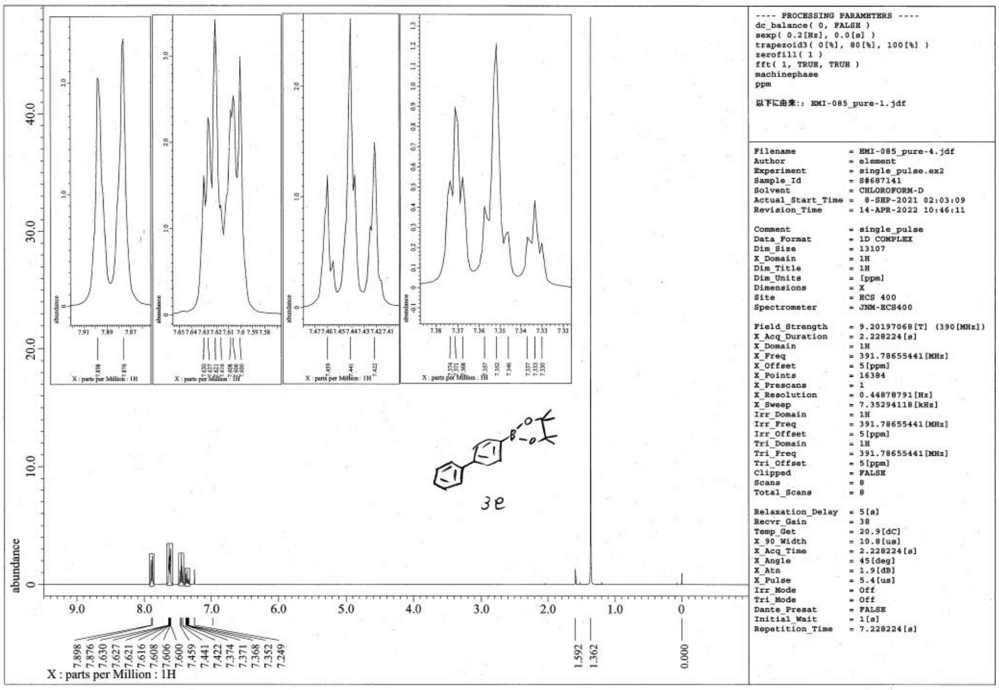

$^{13}$C NMR spectrum of **3e**.

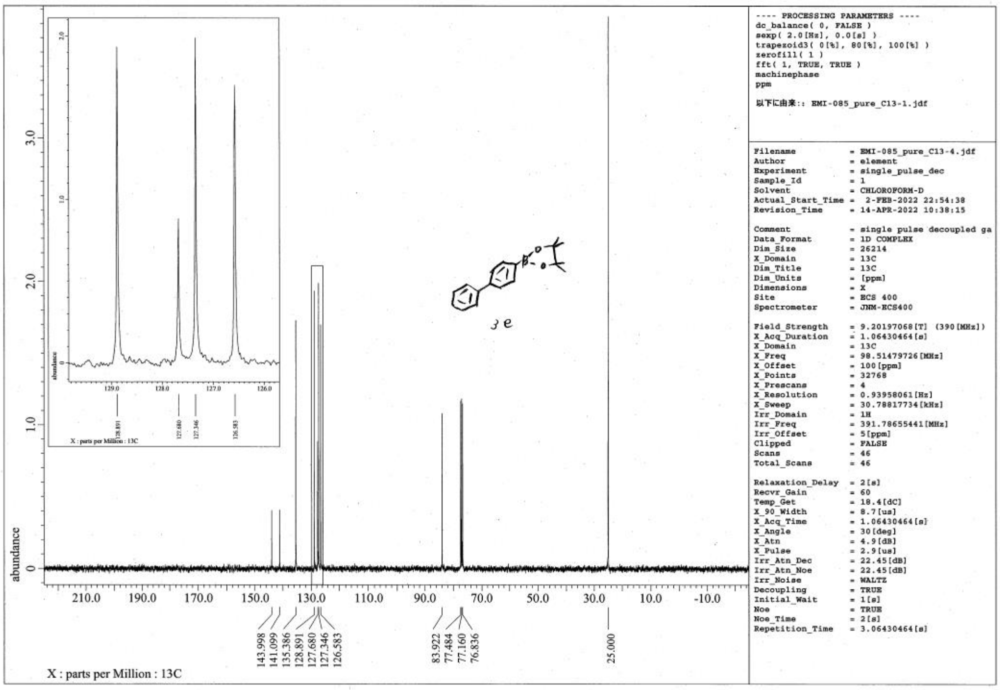

<sup></sup>

$^{1}$H NMR spectrum of **3f**.

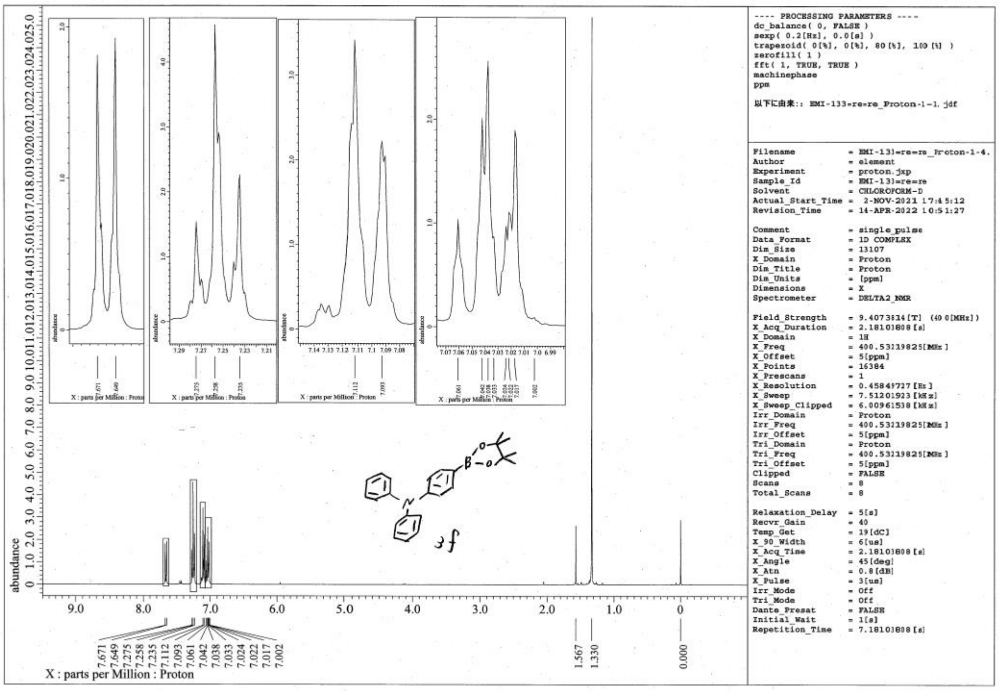

$^{13}$C NMR spectrum of **3f**.

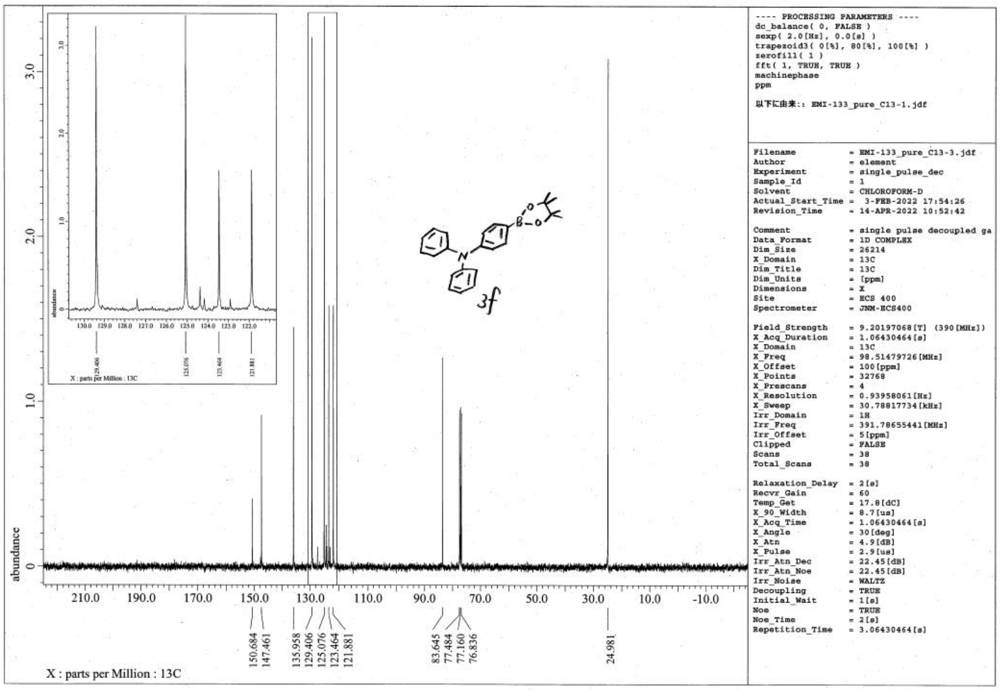

$^{1}$H NMR spectrum of **3g**.

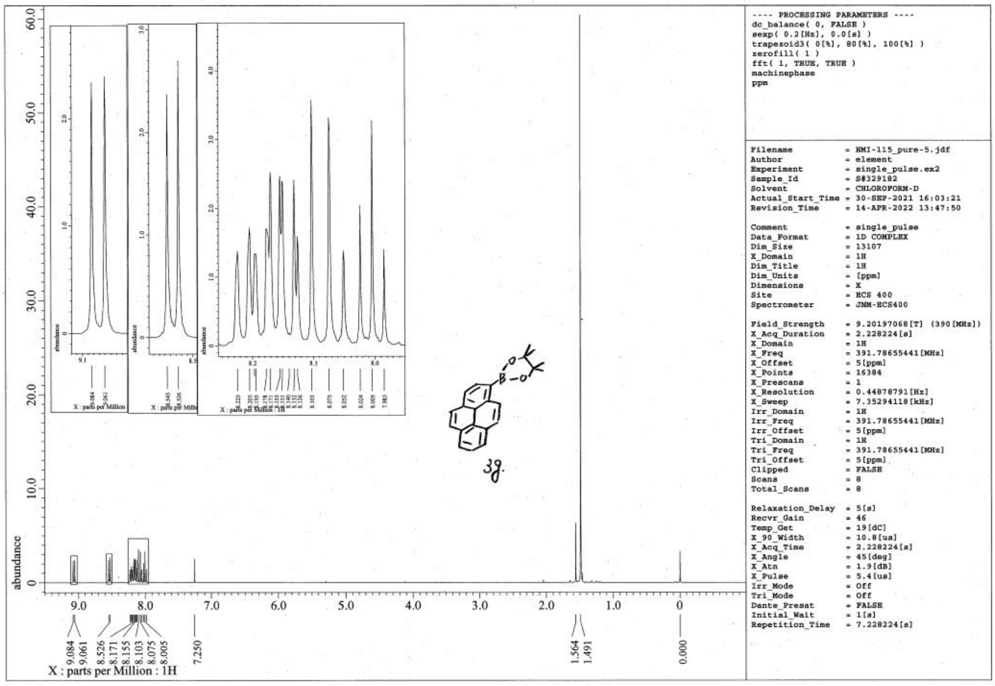

$^{13}$C NMR spectrum of **3g**.

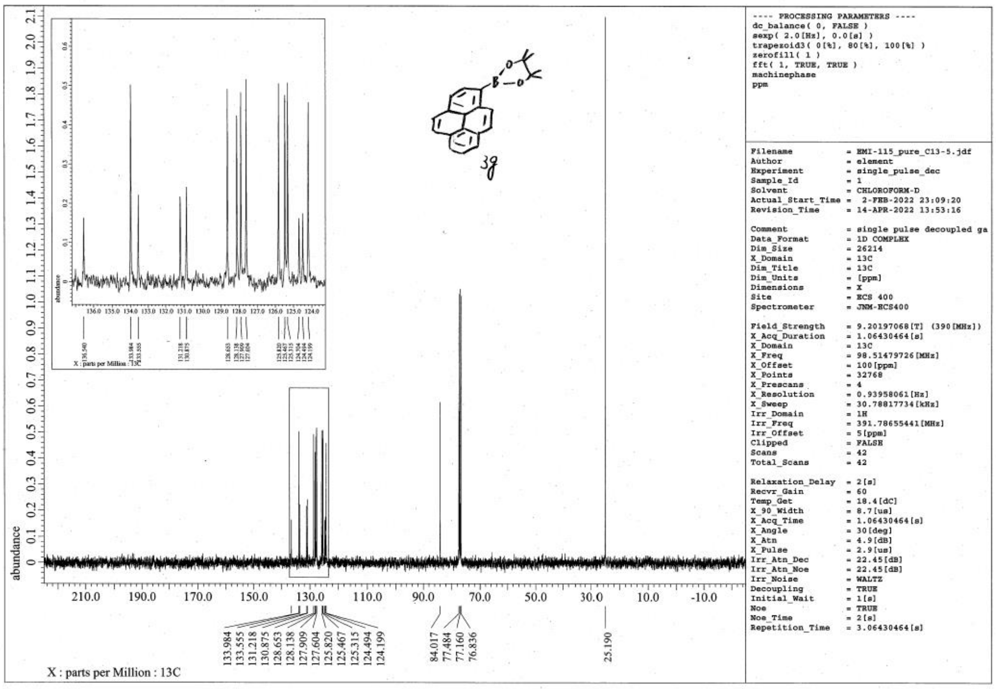

$^{1}$H NMR spectrum of **3h**.

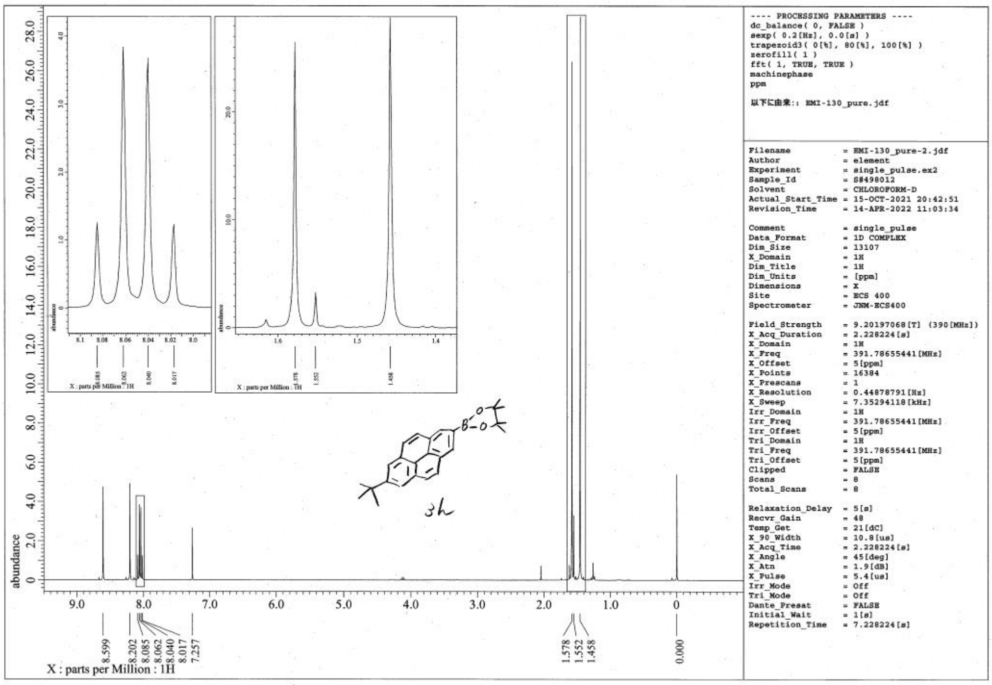


$^{13}$C NMR spectrum of **3h**.

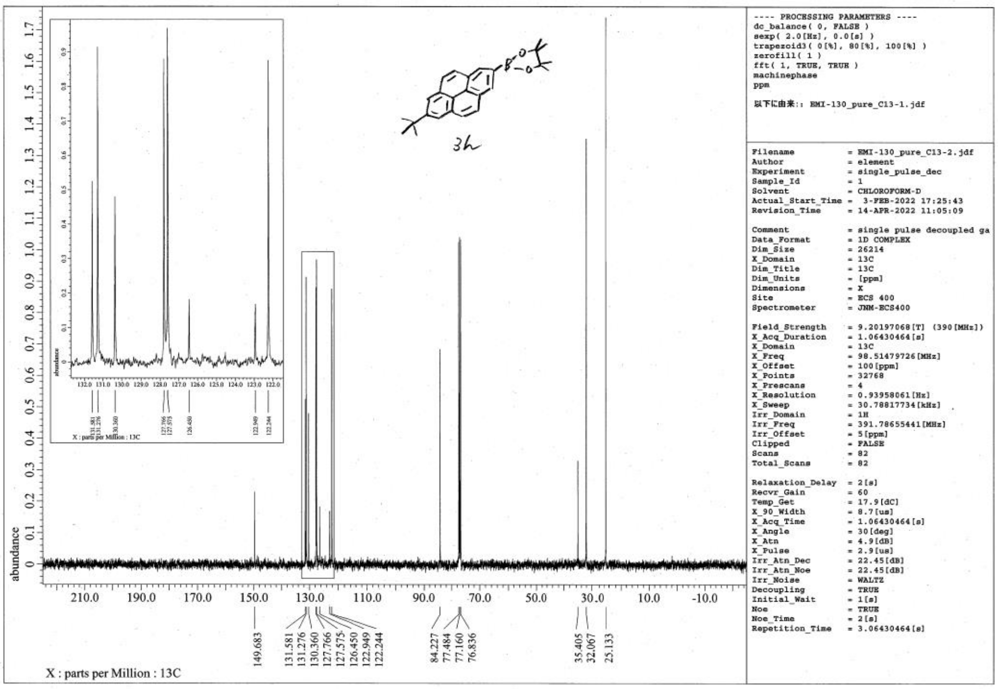

<sup></sup>$^{1}$H NMR spectrum of **3i**.

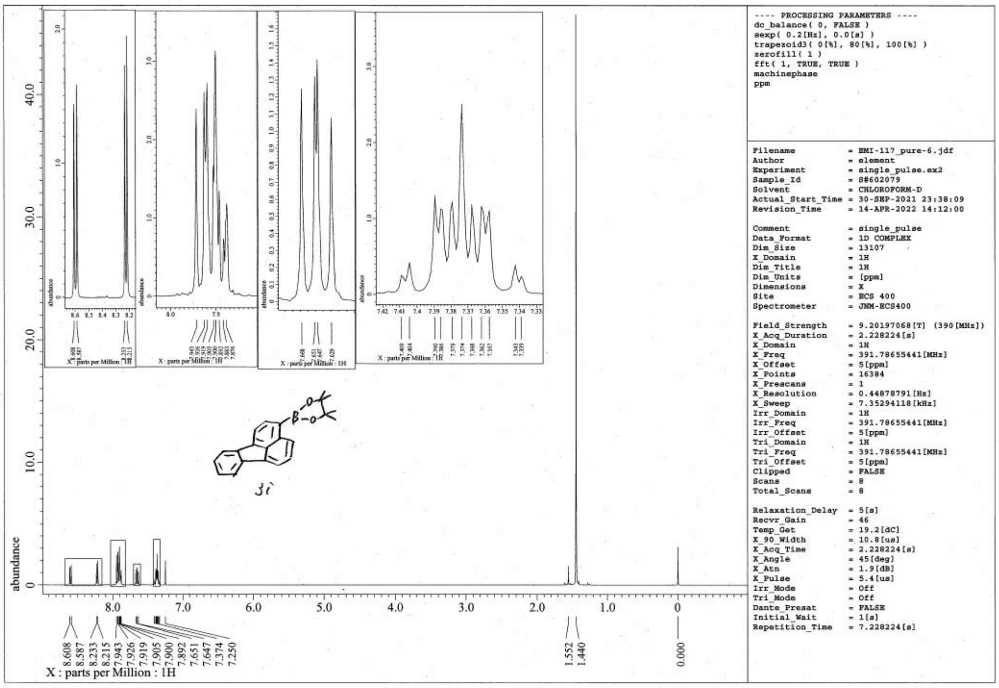

$^{13}$C NMR spectrum of **3i**.

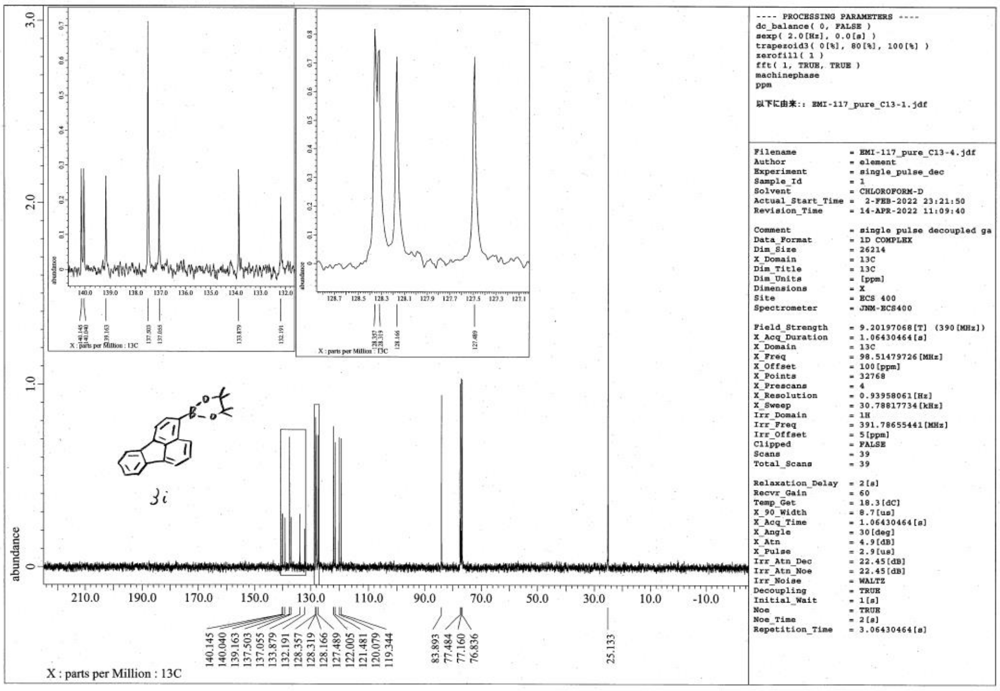

$^{1}$H NMR spectrum of **3j**.

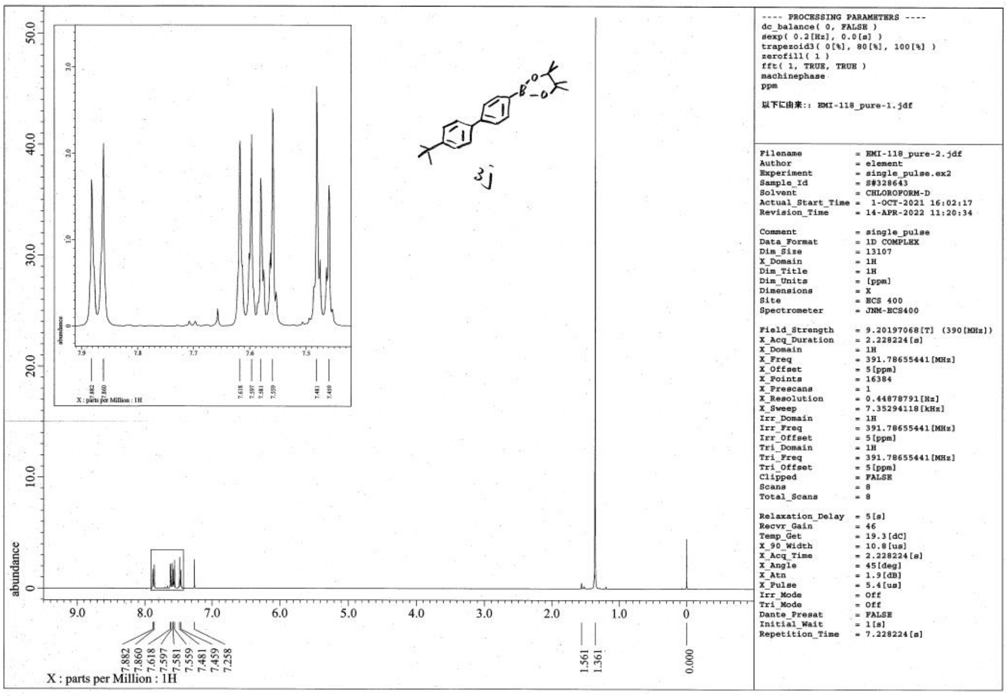

$^{13}$C NMR spectrum of **3j**.

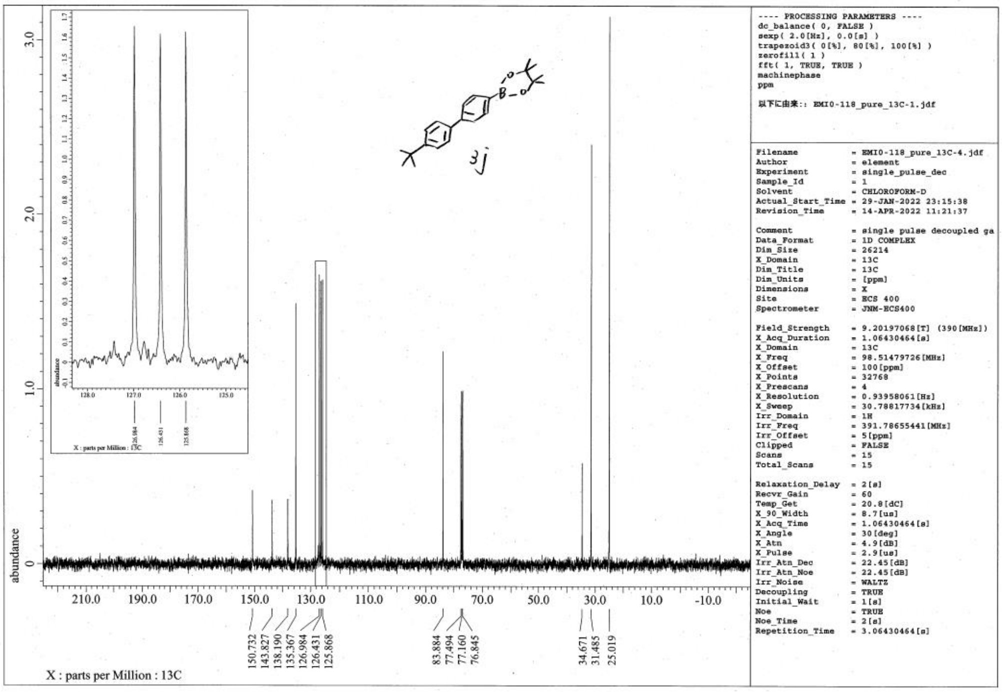

$^{1}$H NMR spectrum of **3k**.

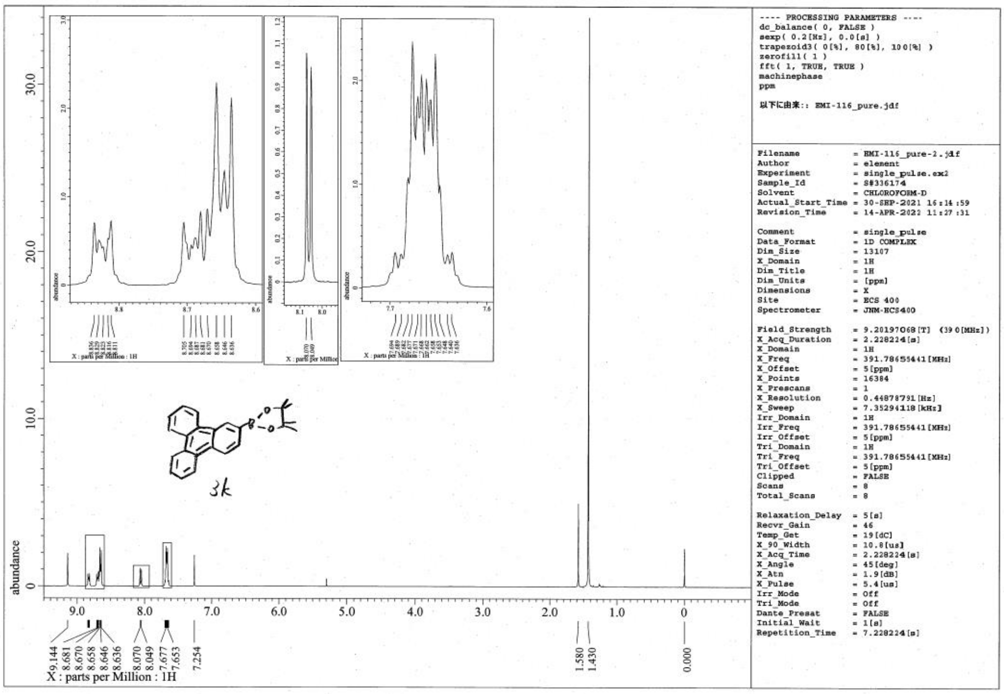

$^{13}$C NMR spectrum of **3k**.

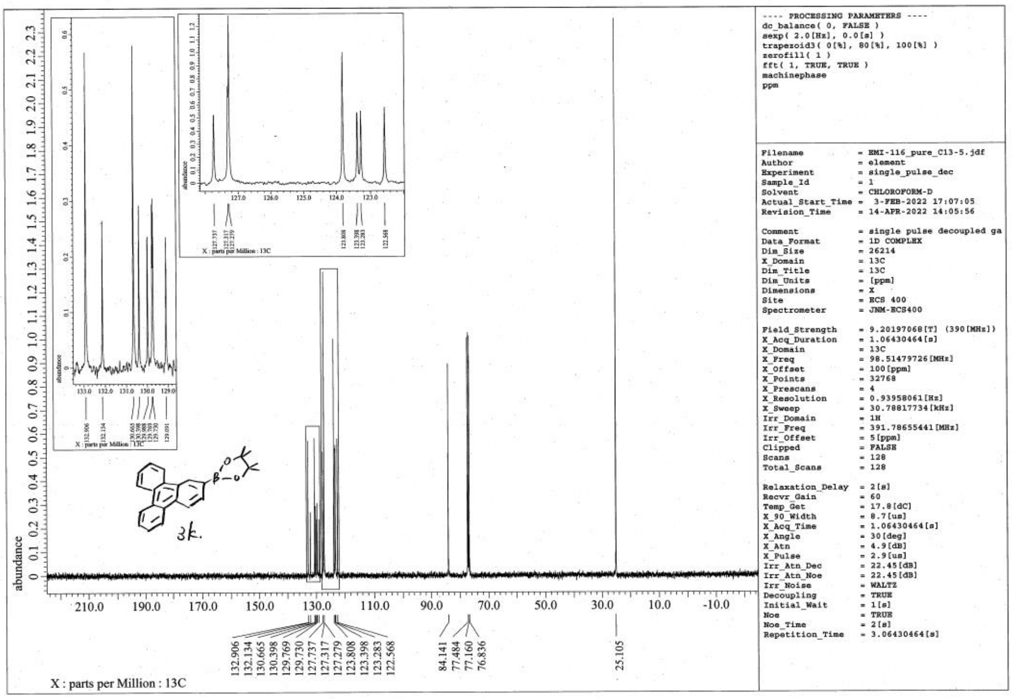

$^1$H NMR spectrum of **3l**.

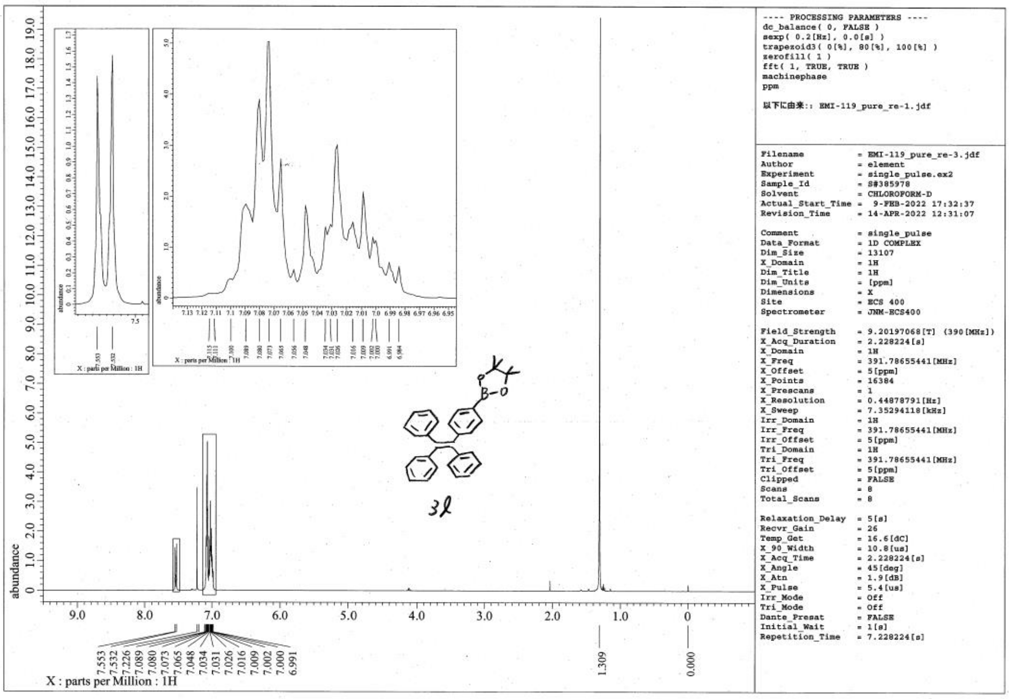


$^{13}$C NMR spectrum of **3l**.

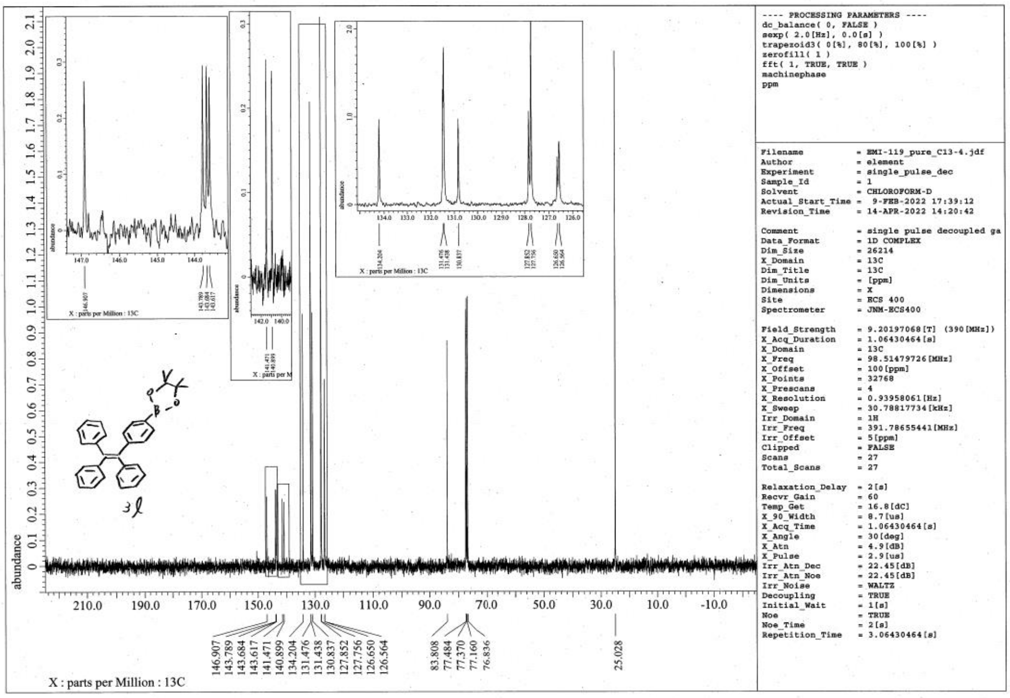

$^{1}$H NMR spectrum of **3m**.

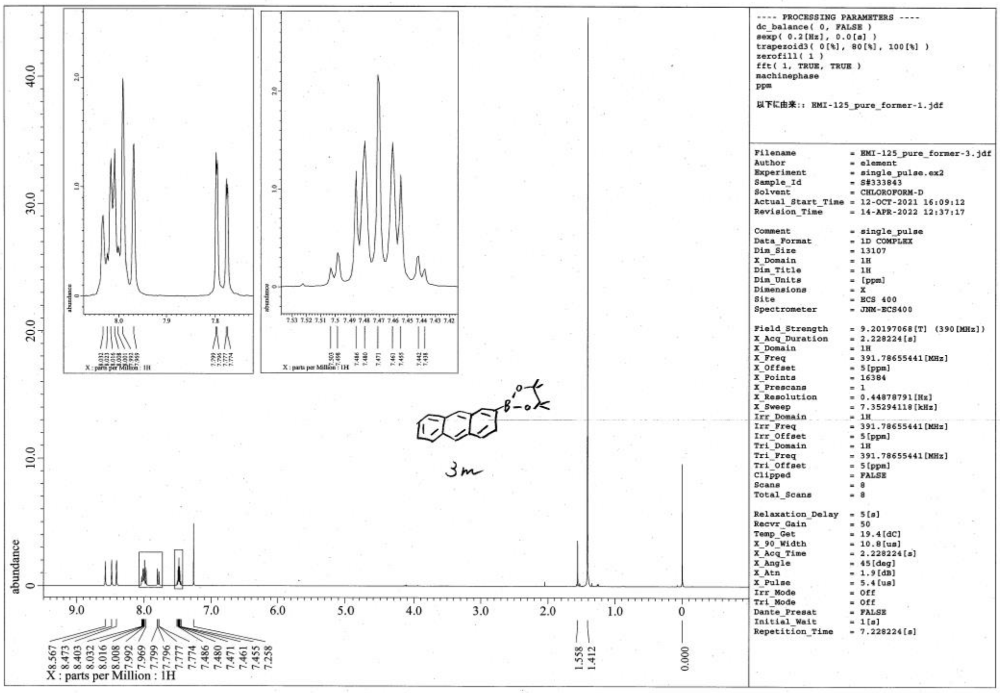

$^{13}$C NMR spectrum of **3m**.

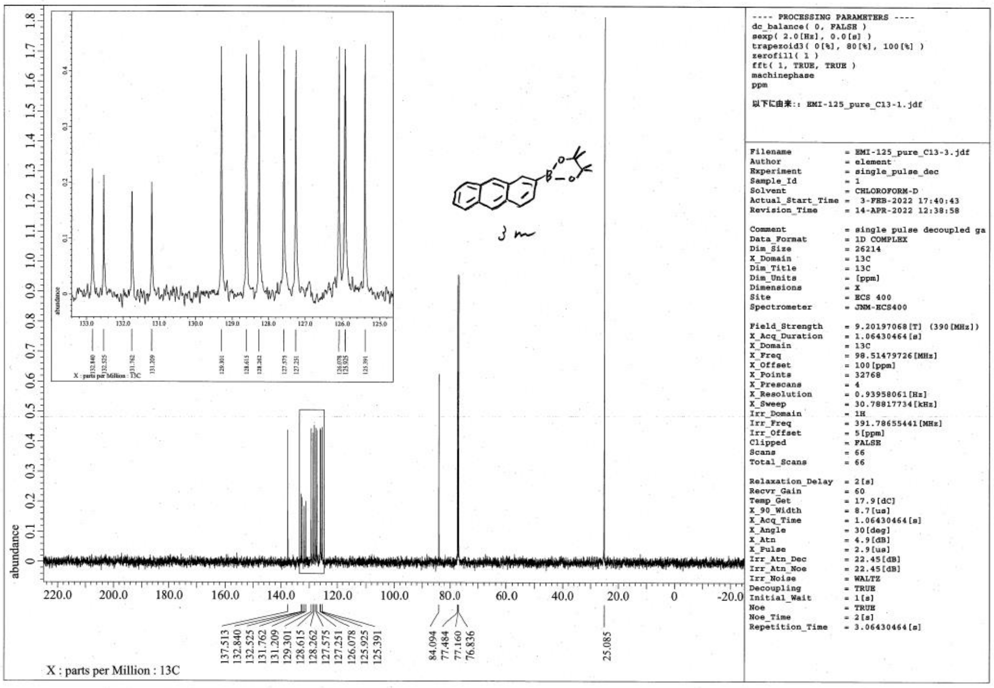

$^{1}$H NMR spectrum of **3n**.

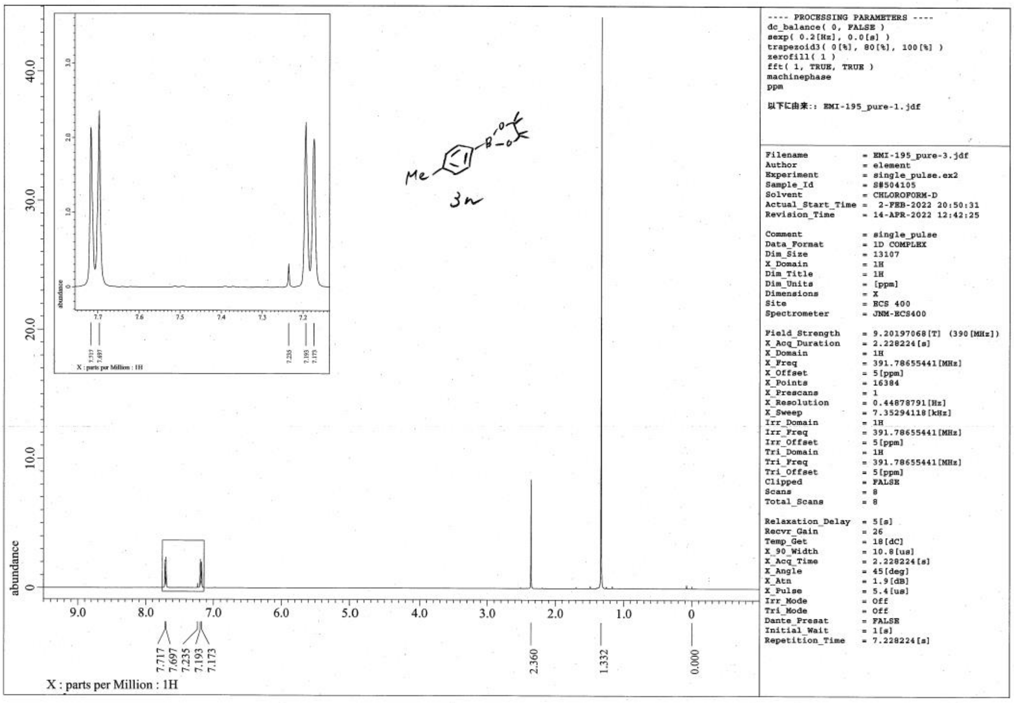

$^{13}$C NMR spectrum of **3n**.

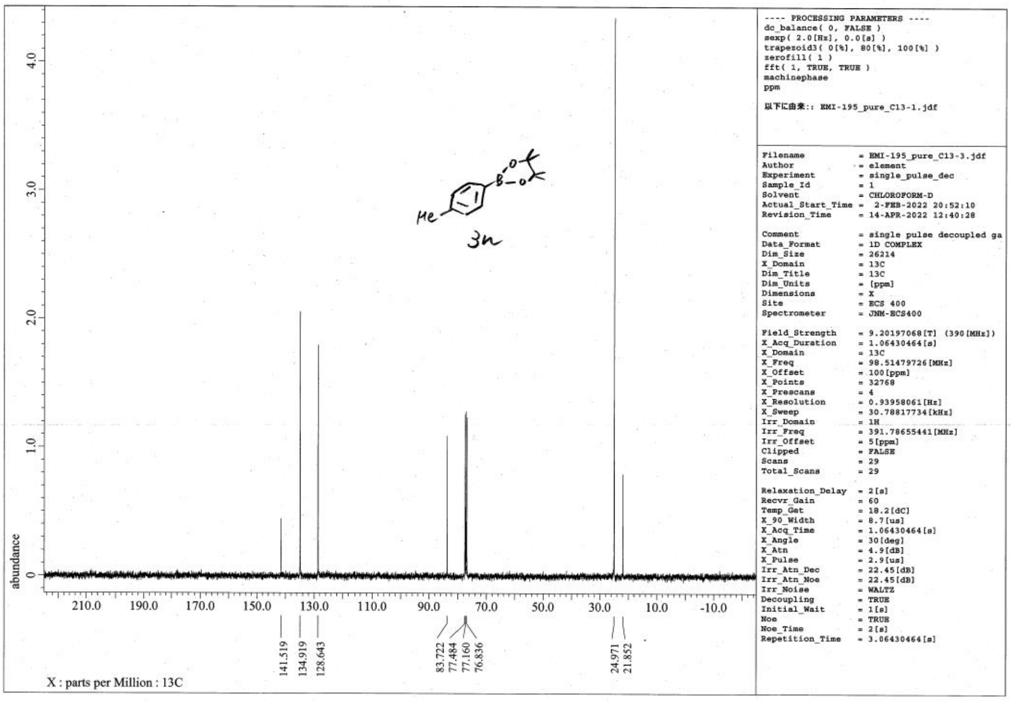

$^{1}$H NMR spectrum of **3o**.

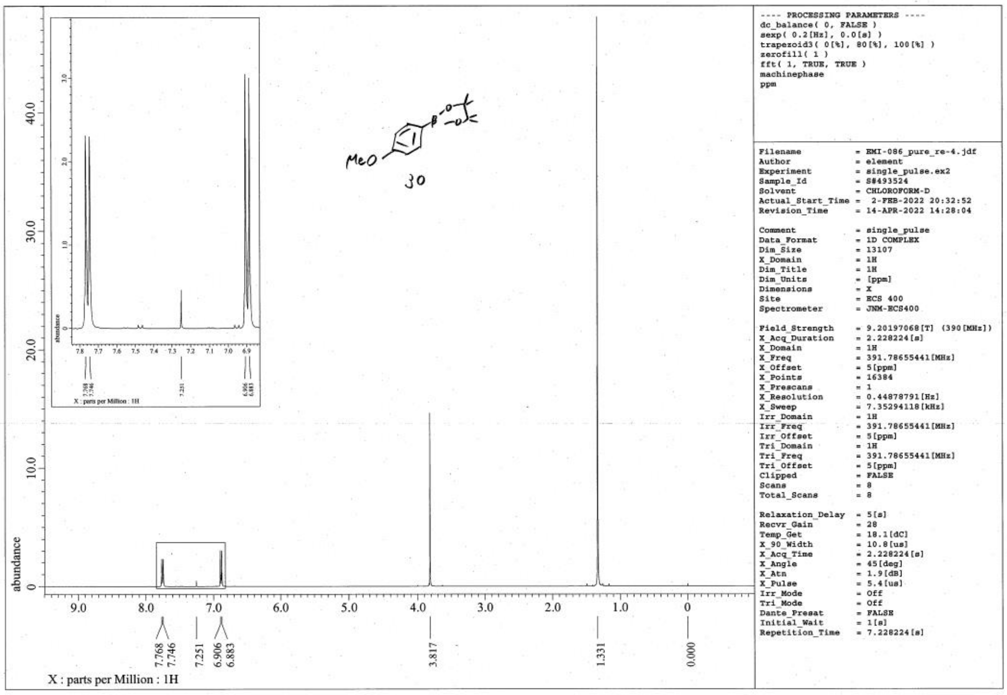

$^{13}$C NMR spectrum of **3o**.

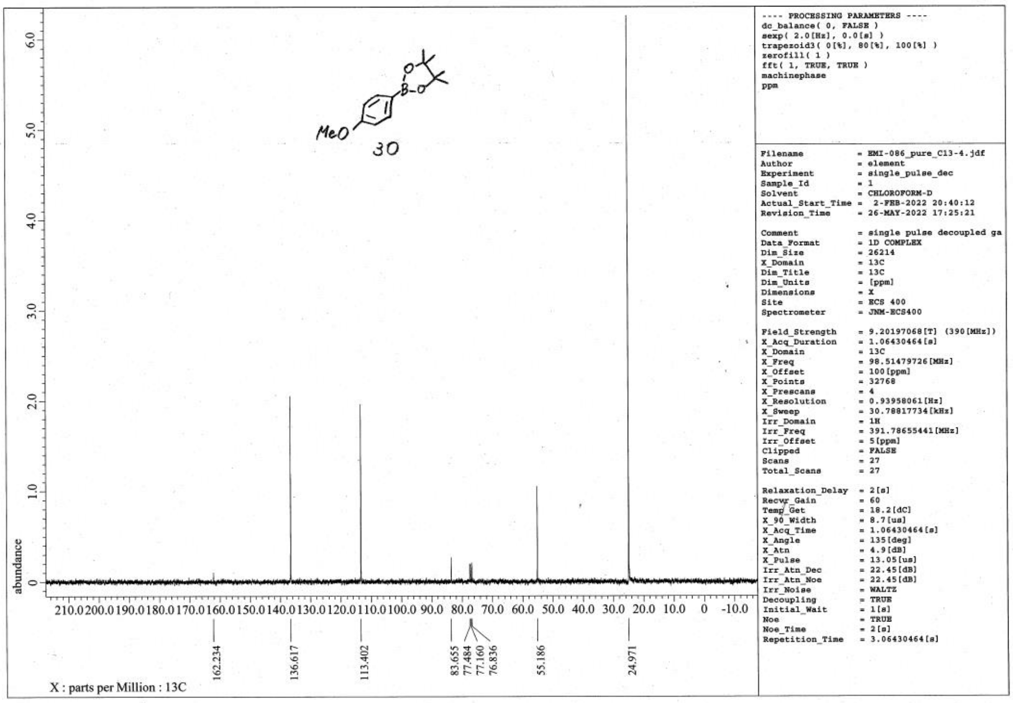

$^{1}$H NMR spectrum of **3p**.

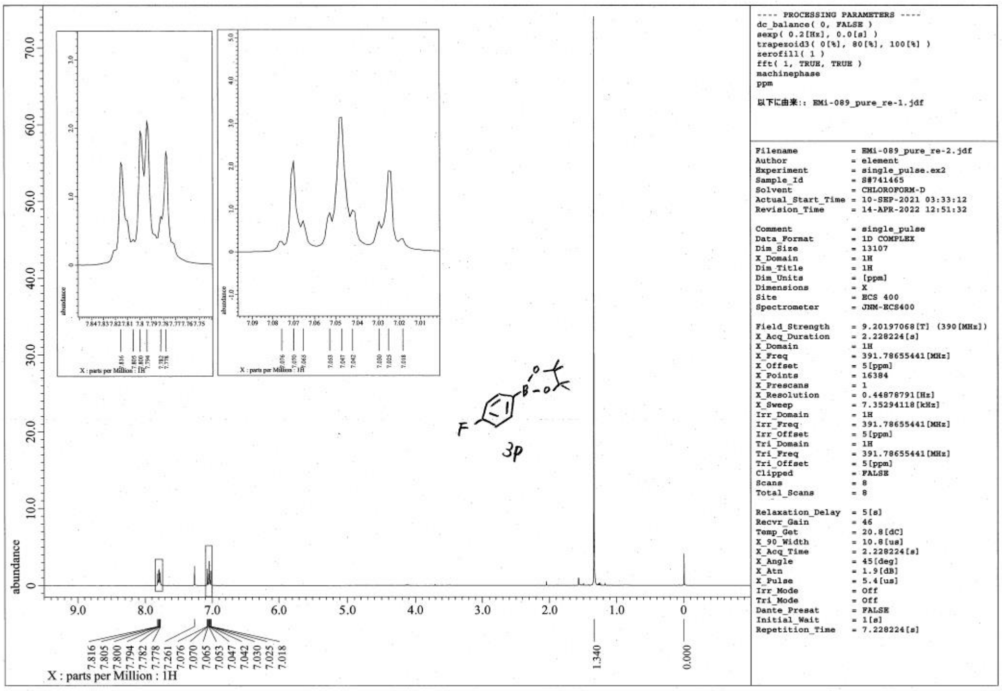

$^{13}$C NMR spectrum of **3p**.

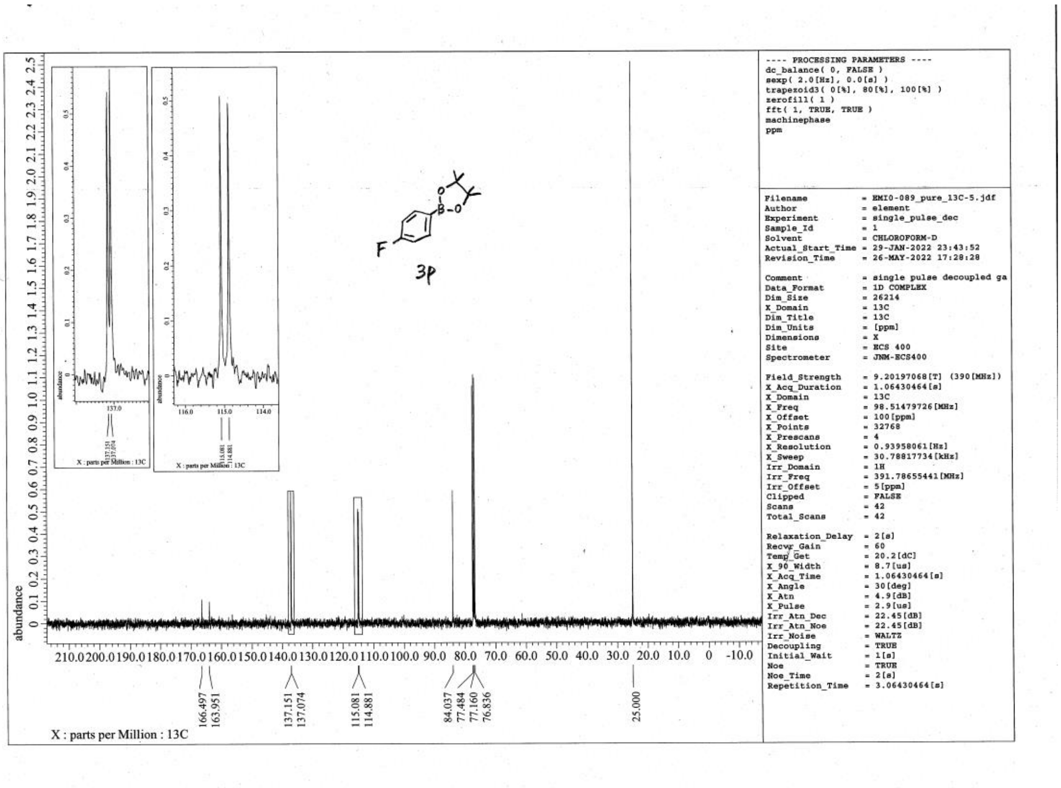

<sup></sup>$^{1}$H NMR spectrum of **3q**.

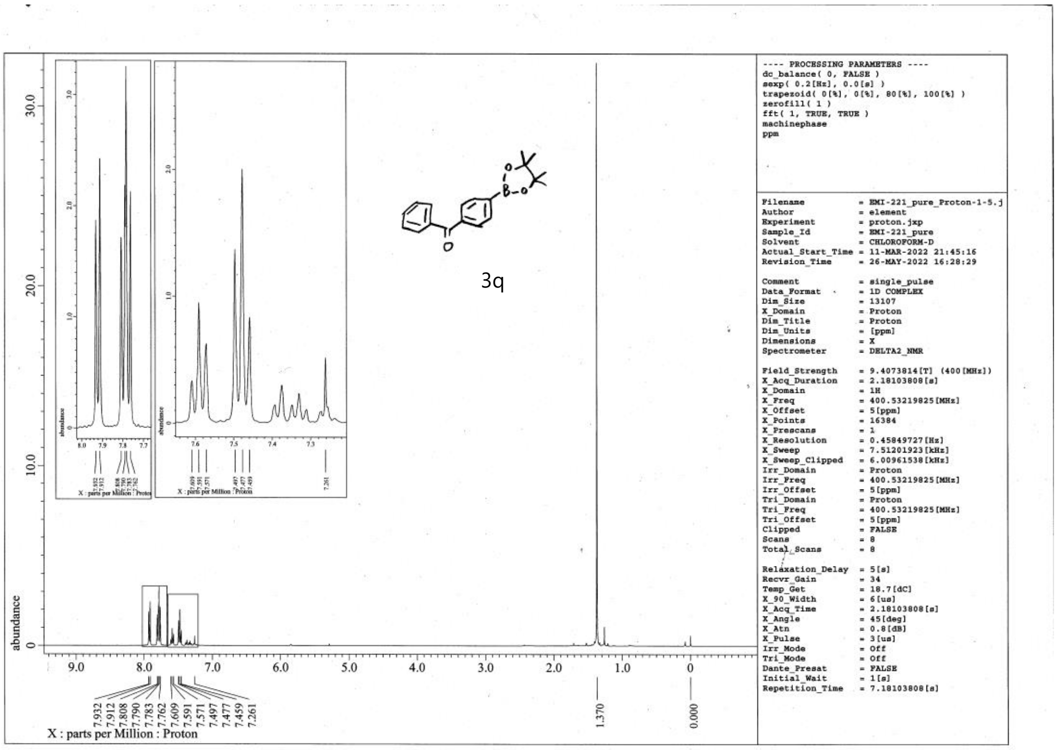

$^{13}$C NMR spectrum of **3q**.

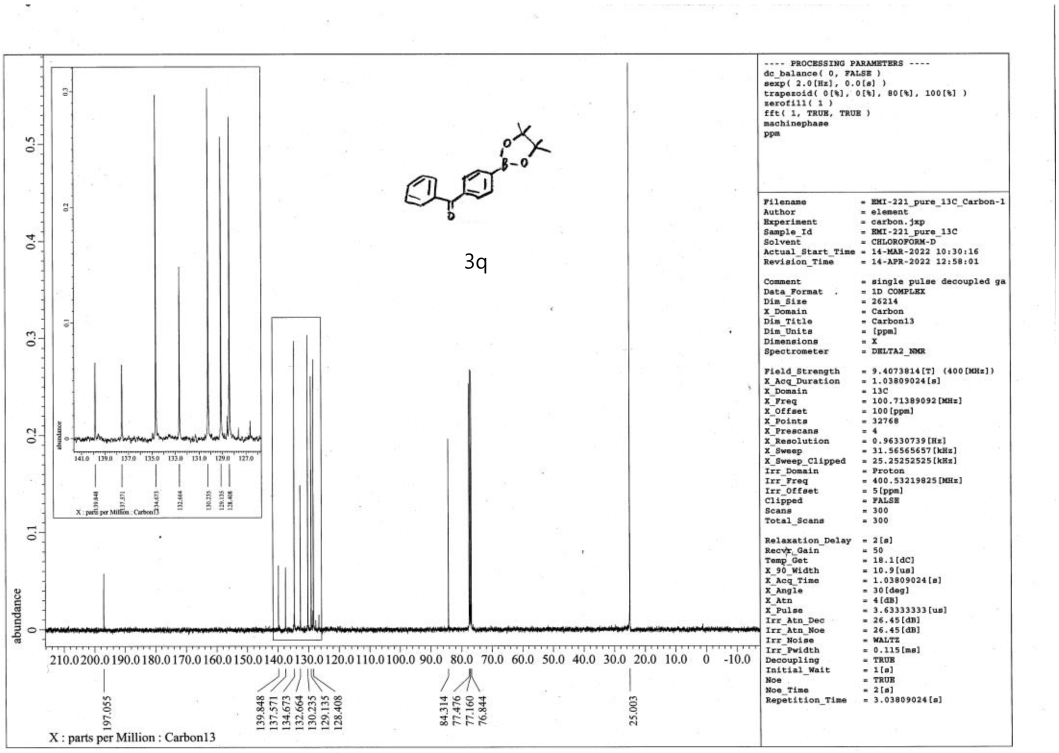

Supplement: File 1 — Experimental procedures, experimental set-ups, characterization data, and NMR spectra. [file Beilstein_J_Org_Chem-18-855-s001.pdf]
